# The Effects of Probiotics Consumption on Blood Pressure, Lipid Profile, Glycemic Indices, and Inflammatory Parameters in Overweight and Obese Adults: A Systematic Review and Meta‐Analysis of Randomized Controlled Trials

**DOI:** 10.1002/fsn3.70434

**Published:** 2025-07-27

**Authors:** Safa Sefidgari‐Abrasi, Marziyeh Rahimiyan‐Heravan, Vahid Amiran, Elham Navval‐Esfahlan, Maryam Saghafi‐Asl

**Affiliations:** ^1^ Student Research Committee Tabriz University of Medical Sciences Tabriz Iran; ^2^ Nutrition Research Center, School of Nutrition and Food Sciences Tabriz University of Medical Sciences Tabriz Iran; ^3^ Faculty of Natural Sciences Middlesex University London UK; ^4^ Department of Molecular Medicine, School of Medicine University of Padua Padua Italy; ^5^ Department of Clinical Nutrition, School of Nutrition and Food Sciences Tabriz University of Medical Science Tabriz Iran

**Keywords:** blood pressure, glucose metabolism, gut microbiota, lipid profile, meta‐analysis, obesity, probiotics

## Abstract

Recent evidence suggests that probiotic supplementation might have beneficial impacts on improving obesity‐related metabolic and inflammatory impairments in animal models and humans. This systematic review and meta‐analysis aimed to review the existing literature about the effects of probiotics on blood pressure, glycemic control, lipid profile, inflammatory factors, and C‐reactive protein levels in people with overweight or obesity. Comprehensive research was conducted across various online databases, including PubMed/MEDLINE, Cochrane, EMBASE, SCOPUS, Web of Science, and ProQuest, as well as ClinicalTrials.gov, ISRCTN, and ICTRP registry platforms. Screening of reference lists was also carried out until March 2023 to identify any relevant randomized controlled trials focusing on the impacts of probiotics on cardiovascular risk factors in obesity, without any language restrictions. The accumulated studies underwent screening and exclusion based on predetermined criteria by four independent researchers. Following this, a meta‐analysis was carried out for the determined groups and subgroups. Finally, the quality of evidence was assessed using the Grading of Recommendations, Assessment, Development, and Evaluation (GRADE) system. Twenty‐six eligible randomized controlled trials, including 1755 overweight and obese adults, were included in the meta‐analysis. Administration of probiotics led to a slight to low‐sized beneficial effect on systolic blood pressure (SBP) (standardized mean difference [SMD] = −0.09; 95% CI: −0.45, 0.26; *p* = 0.605), diastolic blood pressure (DBP) (SMD = −0.27; 95% CI: −0.53, −0.02; *p* = 0.037), fasting plasma glucose (FPG) (SMD = −0.16; 95% CI: −0.26, 0.06; *p* = 0.002), fasting insulin (SMD = −0.22; 95% CI: −0.34, 0.00; *p* = 0.001), total cholesterol (TC) (SMD = −0.10; 95% CI: −0.20, −0.01; *p* = 0.039), Low‐density lipoprotein‐ Cholesterol (LDL‐C) (SMD = −0.11; 95% CI: −0.21, −0.01; *p* = 0.020), High‐density lipoprotein‐ Cholesterol (HDL‐C) (SMD = 0.07; 95% CI: −0.07, 0.20; *p* = 0.316), triglyceride (TG) (SMD = −0.15; 95% CI: −0.27, −0.02; *p* = 0.022), and C‐reactive protein (CRP) (SMD = −0.34; 95% CI: −0.63, −0.05; *p* = 0.020). The findings of the present study indicate that oral supplementation with probiotics yields a small, yet statistically significant beneficial effect on blood pressure, glucose and lipid metabolism, and CRP concentration. However, it's important to note that the present evidence is not conclusive and further large‐scale trials along with additional meta‐analyses are warranted to fully evaluate the potential of probiotics as a new strategy for preventing cardiovascular risk factors in overweight and obesity. It's worth mentioning that this review has been registered on PROSPERO under the registration number CRD42022323003, ensuring transparency and accountability in the research process.

## Graphical abstract

This systematic review and meta‐analysis of 26 randomized controlled trials involving 1755 overweight or obese adults demonstrated that probiotics provide small but statistically significant improvements in diastolic blood pressure, fasting glucose, insulin, total cholesterol, LDL cholesterol, triglycerides, and C‐reactive protein levels. However, changes in systolic blood pressure and HDL cholesterol were minimal or nonsignificant. While these findings highlight the potential cardiovascular and metabolic benefits of probiotics, additional large‐scale studies are required to confirm their efficacy and clinical relevance.

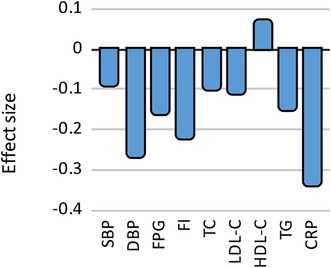

## 1 Introduction

Cardiovascular disease (CVD), as a multifactorial disorder, has long been the leading cause of mortality worldwide. Each year, 17.9 million individuals worldwide die from CVDs, which consist for 32% of all deaths (World Health Organization 2019). It is estimated to affect nearly 23.3 million people by 2030 (Bedani et al. 2015). CVD is mainly linked to obesity, and the risk tends to increase on a continuum with increasing body mass index (BMI) (Thomas et al. 2018). CVD is the direct outcome of incorrect dietary patterns, high systolic blood pressure (SBP), abnormal lipid profile, and high fasting plasma glucose (FPG) levels (Virani et al. 2021).

Metabolic disorders, including hyperglycemia, dyslipidemia, and hypertension, are significant risk factors for cardiovascular diseases and are highly prevalent among overweight and obese individuals. Traditional management of these conditions often includes lifestyle modifications and pharmacological interventions. However, long‐term adherence to such strategies can be challenging due to side effects, cost, and compliance issues. This has spurred interest in adjunctive therapies such as probiotics, which are cost‐effective, accessible, and generally safe. Probiotics have been shown to influence host metabolism through various mechanisms such as modulation of the gut microbiota, reduction of systemic inflammation via increased production of short‐chain fatty acids and modulation of pro‐inflammatory cytokines, and improvement in lipid metabolism and glycemic control through enhanced bile acid metabolism and glucose regulation (Sefidgari‐Abrasi et al. 2021; Dahiya et al. 2017; Mohamadshahi et al. 2014; Hotel and Cordoba 2001). Although several recent clinical trials and meta‐analyses suggested the beneficial impacts of probiotic supplementation on preventing or controlling CVD risk factors including high blood pressure (BP), some studies did not indicate their remarkable effects on CVD risk factors, and the findings are inconclusive (Hariri et al. 2015; Khalesi et al. 2014; Dong et al. 2013; Stenman et al. 2016; Madjd et al. 2016). In addition, several umbrella reviews have explored the effects of probiotics on specific parameters like lipid profiles, glycemic indices, or BP; they often focus on the general population or specific outcomes. Overweight and obese individuals, who are particularly vulnerable to metabolic disorders, have not been adequately represented in such analyses. In addition, there are data from eight trials (Orak et al. 2022; Ben Othman et al. 2023; Choi et al. 2022; Hajipoor et al. 2022; Sohn, Jung, et al. 2022; Cho et al. 2022; Sohn, Na, et al. 2022; Toshimitsu et al. 2021) that have not been considered in previous publications. Furthermore, the umbrella reviews pooled previous meta‐analyses and did not perform meta‐analyses on the original studies. Combining meta‐analyses in umbrella studies increases the risk of double‐counting data and may not lead to accurate results. Despite these earlier studies, the present systematic review and meta‐analysis excluded trials where prebiotics were combined with probiotic capsules, tablets, or sachets, as well as trials involving participants over 65 years old, aiming for more precise results. Additionally, beyond the scope of the previous studies, unpublished papers and gray literature, such as conference papers and dissertations, were also scrutinized in the current paper (Mayta‐Tovalino et al. 2023; Wang et al. 2021, 2019; da Silva Pontes et al. 2021; Yan et al. 2019).

To the best of our knowledge, this systematic review and meta‐analysis represents the first instance where randomized clinical trials incorporating prebiotics, such as fructooligosaccharides (FOS), within the ingredients of placebos, even in minimal quantities, have been excluded. Our objective was to precisely evaluate the impacts of probiotic administration on CVD risk factors—such as SBP and DBP, glycemic indices, lipid profile, and blood levels of pro‐inflammatory cytokines, including C‐reactive protein (CRP) or high‐sensitivity C‐reactive protein (hs‐CRP)—in overweight and obese adults. This study uniquely aims to distinguish the impacts of probiotics from combined interventions, such as FOS or synbiotics, by excluding studies where such distinctions could not be made. Furthermore, this paper stands out by conducting a dose–response analysis to identify the optimal probiotic supplementation dosage and duration for overweight or obese adults.

## 2 Methods

The study adhered to the Preferred Reporting Items for Systematic Reviews and Meta‐Analysis (PRISMA) guidelines (Liberati et al. 2009). The protocol for this study was approved by the Committee of Ethics in Research, Tabriz University of Medical Sciences, Tabriz, Iran (IR.TBZMED.REC.1399.1014). Furthermore, the review was registered on PROSPERO (International Prospective Register of Systematic Reviews) under the registration number CRD42022323003. Although our protocol originally stipulated that databases would be assessed between January 1, 1991, and March 31, 2022, we extended the timeframe until March 31, 2023, to include more recent clinical trials.

### 2.1 Search Strategy

A comprehensive literature search was undertaken for related publications released between January 1, 1991, and March 31, 2023, via the following databases: The Cochrane Central Register of Controlled Trials (CENTRAL), MEDLINE/PubMed, Scopus, Web of Science, EMBASE, ProQuest databases, as well as ClinicalTrials.gov, ISRCTN, and ICTRP registry platforms. It should be mentioned that one of the included trials in the current study was a student thesis discovered through searching gray literature (de Oliveira 2020). No language restrictions or standard filters were used to refine the search.

Keywords of the previous relevant studies, as well as Emtree and MESH indexing systems, were used to find keywords and their synonyms. Broad search strings were used in the literature including “obesity OR overweight” AND “Probiotics”. Full search syntaxes per each database are provided in Table S1.

All reference lists of previously related review articles and RCTs were manually reviewed to identify other relevant trials. Besides, key journals with the highest number of included papers detected by Scopus, including *Nutrient*s, as well as *Food and Function*, were hand‐searched to find other relevant trials and conference papers. In order to avoid any unwanted missing data, ongoing trials were assessed by searching related databases and contacting experts in the field. Unpublished papers and those published in abstract were considered for inclusion only if the required data could be retrieved.

### 2.2 Eligibility Criteria

The findings after applying the search strategies were stored and managed using Mendeley reference manager software (version 1. 19. 8). Duplicated results were removed, and the final library was prepared for further screening.

The primary PICOS criteria (population, intervention, comparison, outcome, study design) were utilized to identify relevant publications. Inclusion criteria for the existing articles were based on the following: (1) Type of study: Original randomized controlled trials (RCTs) with either parallel or crossover designs; (2) Study population: Adults aged between 18 and 65 years who were overweight (BMI: 25–29.9 kg/m^2^) or obese (BMI ≥ 30 kg/m^2^), with or without comorbidities such as diabetes, hypertension, and dyslipidemia; (3) Intervention: Oral supplementation of probiotic species at any dose, duration, and route of administration, either as single or add‐on therapy, excluding combinations with prebiotics as synbiotics; (4) Control: Placebo, lifestyle changes, appetite or absorption suppressants, or no treatment; (5) Outcome: Studies were required to report at least one of the considered outcomes, including BP findings (SBP and DBP), glycemic control (FPG, glycosylated hemoglobin, fasting insulin), lipid profile (total cholesterol (TC), low‐density lipoprotein cholesterol (LDL‐C), high‐density lipoprotein cholesterol (HDL‐C), triglycerides (TG)), inflammatory cytokines, and blood concentration of CRP or hs‐CRP. Articles were screened based on their abstracts or full texts as necessary to ensure they met these inclusion criteria.

The articles were excluded based on the following: (1) Inclusion and exclusion criteria were not declared clearly, (2) Studies did not evidently report outcomes, (3) Supplementation was in combination with prebiotics as a synbiotic, (4) Trials assessing bariatric surgery and fecal transplantation, as experimental intervention or control, (5) Study population younger than 18 or older than 65 years old, and (5) Study participants who were pregnant, lactating, or had any special disease other than diabetes, hypertension, and dyslipidemia. If more than one article was published for one database, the most complete one was considered eligible.

### 2.3 Study Selection

The primary systematic search was undertaken by three reviewers (S.S.‐A., M.R.‐H., and V.A.), followed by a comprehensive selection stage performed by four independent reviewers (S.S.‐A., M.R.‐H., V.A., and E.N.‐E.).

The titles and abstracts of all primary articles that met the search strategy were assessed by three reviewers in the first place to select the eligible studies for inclusion. When a citation was known to be eligible by at least one of the authors or in the case of insufficient title and abstract data, the full text was evaluated. Then, four investigators evaluated the full texts of non‐duplicated articles using a standardized form (S.S.‐A., M.R.‐H., V.A., and E.N.‐E.). In case of disagreements, the discussion was done to reach a consensus. When consensus was not obtained, a fifth reviewer acted as an arbitrator. The between‐reviewer agreement at the full‐text screening stage was assessed and reported as Cohen's kappa coefficient (*κ*).

### 2.4 Data Extraction

A pilot phase of data extraction was considered to standardize authors' knowledge of commonly used terms, train authors in reviewing the data, and discuss the data extraction form. After that, the eligible articles were reviewed by four independent authors (S.S.‐A., M.R.‐H., V.A., and E.N.‐E.) and cross‐checked later. Extracted information included the “mean” and “standard deviation” of outcomes in baseline and post‐intervention.

By applying a standardized data extraction form for further accuracy, the following four major groups of information were collected: (1) Study characteristics (author, journal, year of publication, location, and study design), (2) Baseline characteristics of participants (sample size, age, gender, height, BMI, and body weight), (3) Type of intervention (composition and dosage of supplementation, as well as the duration of intervention), and (4) Data related to outcomes before and after intervention (SBP and DBP, FPG, glycosylated hemoglobin, fasting insulin, lipid profile, inflammatory cytokines, and blood concentrations of CRP or *hs*‐CRP).

In case, data were reported in any form other than means ± standard deviation (SD), they were converted by standard formulas. If data were presented only graphically, they were extracted using a web plot digitizer (https://apps.automeris.io/wpd/) (Rohatgi 2021). Any disagreements were discussed by reviewers and—if required—resolved by consultation with a senior reviewer (M.S.‐A.). If any missing information was observed, it was checked via contact with the corresponding authors by email.

### 2.5 Quality Assessment

Four independent reviewers (S.S.‐A., M.R.‐H., V.A., and E.N.‐E.) assessed the potential bias in the included articles using the Cochrane risk of bias tool for randomized trials (Higgins et al. 2011). Various aspects were considered, including the method of sequence generation, concealment of allocation sequence, blinding of participants and personnel (such as healthcare providers or investigators), blinding of outcome assessors, handling of incomplete outcome data, and selective outcome reporting. Additionally, to enhance the accuracy and precision of the assessment, the reviewers also evaluated the similarity of groups at baseline concerning the most significant prognostic indicators. Each criterion was categorized as having a “low risk of bias,” “unclear risk of bias,” or “high risk of bias,” depending on how it was addressed in each individual study. In cases where there were discrepancies in the scoring among reviewers, consensus was reached through discussion, or the opinion of a fifth author (M.S.‐A.) was sought if necessary.

### 2.6 Data Synthesis and Analysis

The statistical analyses were conducted using Stata statistical software, version 16.0 (Stata Corp, College Station, TX, USA). To ensure consistency across various outcome assessment methods employed in different trials, the SMD was calculated using Glass's delta method to estimate the effect size. Only outcomes reported in at least three eligible studies were included in the meta‐analysis and their SMDs (between baseline and post‐intervention) according to the pre‐registered protocol were pooled using random‐effects analyses due to high methodological heterogeneity (Harris et al. 2008). Given that the random‐effects meta‐analysis already resulted in a nonsignificant overall effect for outcomes with fewer than five studies, we did not perform the Hartung‐Knapp adjustment during the revision stage. We reasoned that applying the Hartung‐Knapp adjustment was unlikely to substantively alter our conclusions. A *p*‐value of < 0.05 was considered statistically significant.

For all studies, data from the last time point were utilized for analysis. In the case of crossover trials, data from the first crossover period were pooled with those from parallel RCTs. If results were presented graphically, the mean ± SD was calculated from the plots.

The standard error (SE) values were converted to SD using the formula: SE × √n, where n represents the sample size. Additionally, median values and their ranges or confidence intervals (CI) were transformed to mean and SD following the method described by Hozo et al. (2005). In instances where study authors did not provide SDs for mean differences, SDs for changes from baseline were estimated using a correlation coefficient calculated in accordance with Cochrane recommendations. This coefficient was applied along with the SDs for baseline and final means for each group to calculate SDs using the formula: SD^2^_change = SD^2^_baseline + SD^2^_final − (2 × correlation coefficient × SD_baseline × SD_final). It is assumed here that the correlation coefficient (*R*) was 0.5 (Higgins 2011).

Heterogeneity among the studies regarding effect measures was evaluated using Cochran's *Q*‐statistics and Higgins *I* ^2^ test (Higgins 2011; Deeks et al. 2019; Higgins et al. 2003). The degree of heterogeneity was categorized as mild (0%–24.9%), moderate (25%–49.9%), severe (50%–74.9%), and highly severe (75%–100%) (Higgins et al. 2003; Higgins and Thompson 2002). If there were at least six trials and an *I* ^2^ value exceeding 25% was observed, the accuracy of data extraction was verified, and potential sources of heterogeneity were explored through subgroup analysis. Subgroup analyses were based on the quality of included trials, type of supplementation (single vs. multiple‐type probiotics), dosage and duration of supplementation, as well as baseline characteristics such as age, gender, health status, weight, BMI, SBP and DBP, glycemic status, and lipid profile. Additionally, a leave‐one‐out removal sensitivity analysis was conducted by systematically omitting trials one by one to assess the impact of individual studies on the overall conclusions and to examine the influence of methodological quality on the robustness of review findings.

Potential publication bias was assessed using funnel plots in the meta‐analysis involving at least ten trials. If asymmetry was observed in the funnel plots, Begg's and Egger's tests were performed (Egger and Smith 1998; Sterne et al. 2001). Furthermore, Duval and Tweedie's trim and fill method was employed if either Begg's or Egger's test yielded a *p*‐value < 0.1 (Duval and Tweedie 2000). A significance level of *p* < 0.05 was utilized for all tests, except for publication bias and meta‐regression tests, where a significance level of *p* < 0.1 was applied.

### 2.7 Subgroup Analysis

A prespecified subgroup analysis was carried out for the main comparison: (1) type of supplementation (supplementation with single vs. multiple‐type probiotics); (2) dosage of intervention (probiotic dosage of < 10^9^, dosage between 10^9^ and 10^10^, and dosage of > 10^10^ cfu); (3) duration of the intervention (interventions lasted for ≤ 8 weeks vs. interventions lasted for > 8 weeks); (4) means of administration (dairy products, sachets or powder, and capsules or tablets); (5) age of participants (individuals < 42 years old vs. individuals ≥ 42 years old); (6) gender (trials on only one sex vs. trials on both sexes); (7) health status (trials on healthy participants vs trials on participants with comorbidities including hypertension, diabetes, or dyslipidemia); (8) BMI (trials with mean BMI between 25 and 29.9 kg/m^2^ vs. trials with mean BMI equal to or above 30 kg/m^2^).

Additionally, a subgroup analysis was carried out according to the risk of bias. Studies were classified into three categories: “low,” “moderate,” and “high” risk of bias. Trials deemed to have a low overall risk of bias were those assessed as low risk in key domains such as sequence generation, blinding of participants and personnel, and incomplete outcome data. Trials categorized as having a high overall risk of bias were those rated as high or unclear risk in two or three of these domains. Trials with a high or unclear risk of bias in just one key domain were considered to have a moderate risk of bias.

Moreover, meta‐regression analysis was executed based on probiotic dose and duration of intervention to find out an optimum probiotic supplementation dosage and duration. A *p*‐value < 0.1 was considered statistically significant.

### 2.8 Quality of Evidence

We used the GRADE (Grading of Recommendations, Assessment, Development, and Evaluation) system in order to classify the quality of evidence generated by this meta‐analysis, and the quality was classified as high, moderate, low, or very low (Guyatt et al. 2011). This classification system helps to evaluate the confidence level in the data presented in the meta‐analysis, thus providing insights into the reliability and robustness of the findings.

The evidence quality of a meta‐analysis was initially considered high. However, it could be downgraded if certain factors were present, such as study methodological limitations (e.g., when most included trials exhibit an overall high risk of bias), inconsistency among the results of individual trials, imprecision of estimates of effect (indicating unacceptable random error), and evidence of publication bias (e.g., favoring studies with positive results).

Conversely, the quality of evidence could be upgraded if certain factors were observed, such as a magnitude of effect so large that bias common to observational studies cannot possibly account for the result, a dose–response relationship indicating a proportional result to the degree of exposure, and control of all plausible confounders, suggesting that the observed effect is likely conservative.

## 3 Results

### 3.1 Search Results and the Characteristics of Included Trials

**FIGURE 1:**
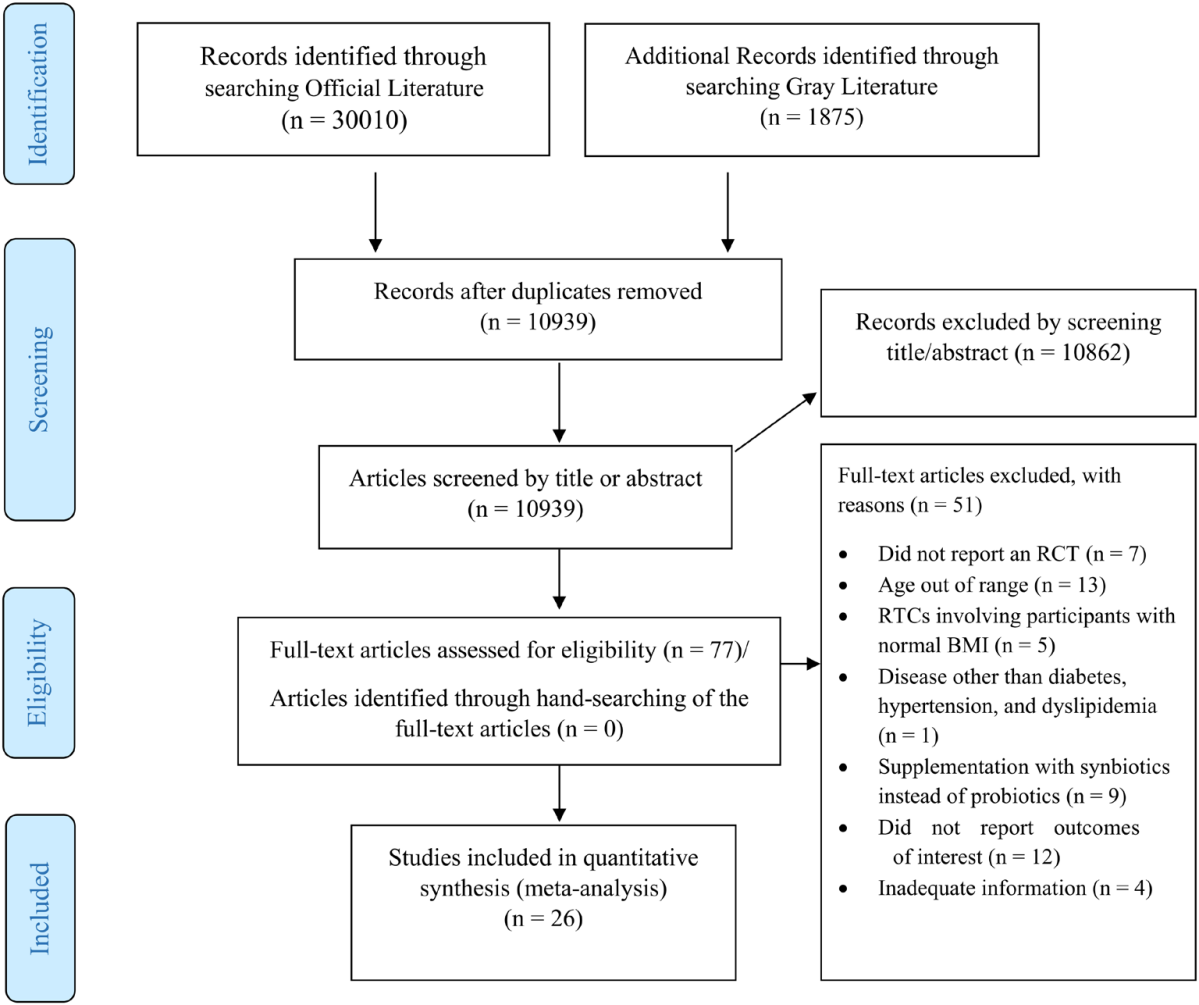
PRISMA flow diagram of the study selection process.

A total of 31,885 records (5003 from PubMed, 7207 from Scopus, 12,289 from Web of Science, 5962 from Embase, 1152 from CENTRAL, 261 from ProQuest, 5 from ClinicalTrials.gov, and 6 from ICTRP) were detected, from which 20,946 were excluded after duplicate deletion (Supp. Table 1). Three authors identified 83 articles after screening the titles and abstracts of 10,939 records, from which 6 were removed due to lack of access to the full texts. Of the 77 studies, 51 did not match the eligibility criteria. Finally, 26 eligible trials were included in the present systematic review and meta‐analysis (Figure 1). The between‐reviewer agreement for including studies was 0.86 at the full‐text screening step. A total of 1755 overweight and obese individuals were randomized into probiotic (*n* = 883) and control (*n* = 872) groups. The mean age of the participants in these trials ranged from 32 to 48.5 years. The summarized characteristics of these 26 eligible articles are presented in Table 1. Probiotics were supplemented in different forms including kashk (Razmpoosh et al. 2020), fermented milk or yogurt (Madjd et al. 2016; Hajipoor et al. 2022, 2021; Toshimitsu et al. 2021; Agerholm‐Larsen et al. 2000; Naito et al. 2017; Zarrati et al. 2014, 2013), powder or sachets (Stenman et al. 2016; Gomes et al. 2017; Jung et al. 2015; Krumbeck et al. 2018; Rahayu et al. 2021), and capsules or tablets (Orak et al. 2022; Ben Othman et al. 2023; Choi et al. 2022; Sohn, Jung, et al. 2022; Cho et al. 2022; Sohn, Na, et al. 2022; de Oliveira 2020; Lim et al. 2020; Nasiri et al. 2020; Schellekens et al. 2021; Déchelotte et al. 2021; Rajkumar et al. 2014). The duration of the probiotic administration ranged from 3 (54) to 26 (22) weeks.

**TABLE 1:** Characteristics of included randomized controlled trials.

First author, year	Design	Country	Gender	Mean age	Health status	Participants (intervention, control)	Duration in weeks	Intervention (dosage and probiotic strains)	Control status	Outcomes
Agerholm‐Larsen et al. (2000)	Parallel double‐blind randomized controlled clinical trial	Denmark	Both	38.5	Pre‐hypotensive	16, 14	8	Daily 450 mL of the test yogurt containing one strain of *Enterococcus faecium* (6 × 10^7^ CFU/mL) and two strains of *S. thermophilus* (10^9^ CFU/mL)	Daily 450 mL of placebo yogurt	SBP, DBP, TC, LDL, HDL, TG
Cho et al. (2022)	Parallel double‐blind randomized controlled clinical trial	Korea	Both	44	Prehypertensive, Predyslipidemic, and Prediabetic	38, 37	12	500 mg of MED‐02 probiotic capsule once daily, containing two strains of *L. fermentum MG4231* and *MG4244*, each at 2.5 × 10^9^ CFU	Daily one placebo capsule containing maltodextrin	SBP, DBP, TC, LDL, HDL, TG, FPG, *hs*‐CRP
Choi et al. (2022)	Parallel double‐blind randomized controlled clinical trial	Canada	Both	33.5	Healthy	69, 63	12	Daily use of one probiotic capsule containing *L. rhamnosus* HA‐114, (10 × 10^9^ CFU per capsule)	Appearance‐ and taste‐matched placebo capsule, once daily	SBP, DBP, TC, LDL, HDL, TG, FPG, fasting serum insulin, CRP
Crovesy de Oliveira (2020)	Parallel double‐blind randomized controlled clinical trial	Brazil	Female	33	Prehypertensive	10, 11	8	Daily one capsule of 10^9^ CFU *B. lactis* and a sachet containing 5 g of maltodextrin	Daily one capsule containing gelatin and a sachet containing 5 g of maltodextrin	SBP, DBP, TC, LDL, HDL, TG, FPG
Déchelotte et al. (2021)	Parallel double‐blind randomized controlled clinical trial	France	Both	NI	Prehypertensive and Predyslipidemic	104, 108	12	Probiotic capsule twice a day, containing *Hafnia alvei* HA4597 strain, 10^10^ CFU per day	Placebo capsule twice a day	TC, LDL, HDL, FPG, HbA1c
Gomes et al. (2017)	Parallel double‐blind randomized controlled clinical trial	Brazil	Female	42	Healthy	21, 22	8	Daily four sachets of probiotic mix containing 2 × 10^10^ CFU of probiotic strains including two strains of *Lactobacilli*, two strains of *Bifidobacteria*, and *Lactococcus lactis* LL‐23	Daily four placebo sachets, similar to the active product in appearance, smell, and taste	HbA1c, TC, LDL, HDL, TG, TNF‐α, IL‐6, IL‐10
Hajipoor et al. (2021, 2022)	Parallel double‐blind randomized controlled clinical trial	Iran	Both	38	Predyslipidemic	28, 31	10	Daily intake of 100 g of probiotic yogurt cantaining *L. acidophilus La‐B5* and *B. lactis Bb‐12* (at levels of 4 × 10^7^ CFU of each strain)	Daily intake of 100 g regular low‐fat yogurt	FBS, fasting serm insulin TC, LDL, HDL, TG
Jung et al. (2015)	Parallel double‐blind randomized controlled clinical trial	Korea	Both	39	Healthy	49, 46	12	2 g of powder containing *L. curvatus* HY7601 and *L. plantarum* KY1032, each at 2.5 × 10^9^ CFU, twice a day	2 g of placebo powder containing crystalline cellulose, lactose, and blueberry‐flavoring agent, twice a day	SBP, DBP, FPG, fasting serum insulin, TC, LDL, HDL, TG, *hs*‐CRP
Krumbeck et al. (2018)	Parallel double‐blind randomized controlled clinical trial	USA	Both	44	Prehypertensive	14, 17	3	Daily one sachet of 0.1 g *B. adolescentis* IVS‐1 powder (10^10^ CFU/g), resulting in a daily dose of 10^9^ CFU and 6.9 g of lactose as a carrier/control	Daily one placebo sachet containing 7 g of lactose	SBP, DBP, FPG, TC, LDL, HDL, TG
Lim et al. (2020)	Parallel double‐blind randomized controlled clinical trial	Korea	Both	47	Prediabetic	57, 57	12	Daily two allocations of 5 × 10^9^ CFU of *L. sakei* (CJLS03)	Daily two allocations of the equivalent vehicle	SBP, DBP, FPG, HbA1c, fasting serum insulin, TC, LDL, HDL, TG
Madjd et al. (2016)	Parallel single‐blind randomized controlled clinical trial	Iran	Female	32	Healthy	44, 45	12	Daily 400 g of probiotic yogurt, enriched with 10^7^ CFU of two strains of *Lactobacilli* and *Bifidobacteria*	Daily 400 g of low‐fat yogurt; no difference in composition, color, taste, and texture with the probiotic yogurts	FPG, HbA1c, fasting serum insulin, TC, LDL, HDL, TG
Naito et al. (2017)	Parallel double‐blind randomized controlled clinical trial	Japan	Male	47	Prediabetic	48, 50	8	One 100‐mL bottle of fermented milk containing *LcS‐*fermented milk containing > 10^11^ CFU of *L. casei* strain Shirota YIT 9029	One 100‐mL bottle of non‐fermented placebo milk	SBP, DBP, FPG, HbA1c, fasting serum insulin, TC, LDL, HDL, TG
Nasiri et al. (2020)	Parallel double‐blind randomized controlled clinical trial	Iran	Both	34.5	Prehypertensive	21, 21	8	500 mg probiotic capsules: containing 2 × 10^11^ CFU of probiotic strains including four strains of *Lactobacilli*, two strains of *Bifidobacteria*, and *S. thermophilus*	500 mg of placebo capsules, once a day	SBP, DBP, CRP
Orak et al. (2022)	Parallel single‐blind randomized controlled clinical trial	Turkey	Female	32	Healthy	17,17	8	Probiotic tablets twice a day, each containing various probiotic strains in the amount of 1.5 × 10^9^ cfu/g in each capsule, including *E. faecium, L. plantarum, S. thermophilus, B. lactis, L. acidophilus*, and *B. longum* .	A weight‐loss diet program and an exercise program without probiotic supplementation	FBS, TG, TC, LDL, HDL, fasting serum insulin
Ben Othman et al. (2023)	Parallel single‐blind randomized controlled clinical trial	Tunisia	Both	48.5	Healthy	15, 15	4	One tablet containing four microbiological strains: *B. longum, L* *. helveticus* , *L* *. lactis* , *and S. thermophilus* (10.10^9^ CFU/capsule/day)	A low‐carbohydrate and low‐calorie diet without any intervention	SBP, DBP, TC, TG, HDL, LDL, FBS, fasting serum insulin, HbA1c
Rahayu et al. (2021)	Parallel double‐blind randomized controlled clinical trial	Indonesia	Both	44	Healthy	30, 30	12	One sachet of one g of skimmed milk powder containing *L. plantarum Dad‐13* (2 × 10^9^ CFU in each packing) once a day	One sachet of one g of skimmed milk powder without probiotics	TC, TG, LDL, HDL
Rajkumar et al. (2014)	Parallel randomized controlled clinical trial	India	Both	NI	Healthy	15, 15	6	One capsule daily containing 112.5 × 10^9^ CFU of three strains of *Bifidobacteria*, four strains of lactobacilli, and one strain of *S. salivarius* subsp. thermophilus	One placebo capsule daily containing 40 mg microcrystalline cellulose	FPG, fasting serum insulin, TC, LDL, HDL, TG, CRP
Razmpoosh et al. (2020)	Parallel randomized controlled clinical trial	Iran	Female	36	Healthy	32, 33	8	Low‐calorie diet and 50 g of kashk containing 1.85 × 10^6^ CFU/g of *L. acidophilus* La5 and 1.79 × 10^6^ CFU/g of *B. lactis* Bb12	Low‐calorie diet	SBP, DBP, FPG, TC, LDL, HDL, TG
Schellekens et al. (2021)	Parallel double‐blind randomized controlled clinical trial	Ireland	Both	45	Healthy	74, 48	12	Daily one capsule, provides a daily dose of 10^10^ CFU of *B. longum* APC1472	Daily one placebo capsule containing maltodextrin	FPG, HbA1c, fasting serum insulin, TC, LDL, HDL, TG, TNF‐α, IL‐10, IFN‐γ
Sohn, Jung, et al. (2022)	Parallel double‐blind randomized controlled clinical trial	Korea	Both	43	Prehypertensive	35, 36	12	Two daily allocations of 2 × 10^9^ CFU of LPK (total 4 × 109 CFU/day)	Placebo capsules twice a day	SBP, DBP, TC, TG, HDL, LDL, FPG, fasting serum insulin, TNF‐α, IL‐6, *hs*‐CRP
Sohn, Na, et al. (2022)	Parallel double‐blind randomized controlled clinical trial	Korea	Both	40	Prehypertensive	50, 49	12	Daily one capsule containing 1 × 10^10^ CFU of *L. plantarum* (LMT1‐48)	Daily one placebo capsule containing maltodextrin	SBP, DBP, TC, TG, HDL, LDL, FBS, fasting serum insulin, *hs*‐CRP
Stenman et al. (2016)	Parallel double‐blind randomized controlled clinical trial	Finland	Both	48.5	Healthy	25, 36	26	One microcrystalline sachet per day containing 12 g of cellulose and *B. animalis* ssp. lactis 420 (10^10^ CFU/day)	One microcrystalline sachet per day containing 12 g of cellulose	IL‐6, *hs*‐CRP
Toshimitsu et al. (2021)	Parallel double‐blind randomized controlled clinical trial	Japan	Both	45	Predyslipidemic	46, 46	12	112 g test yogurt daily, containing > 5 × 10^9^ CFU heat‐treated *L. plantarum* OLL2712	112 g placebo yogurt daily	SBP, DBP, TC, LDL, HDL, TG, FPG, HbA1c, fasting serum insulin, IL‐6, IL‐8, TNF‐α, *hs*‐CRP
Zarrati et al. (2013, 2014)	Parallel double‐blind randomized controlled clinical trial	Iran	Both	35.5	Healthy	25, 25	8	Probiotic yogurt containing 10^8^ CFU/mL of each *S. thermophiles* and *L. bulgaricus* strain	Conventional yogurt containing only starter cultures of *S. thermophilus* and *L. bulgaricus*	*hs*‐CRP SBP, DBP, TNF‐α, IL‐4, IL‐10, IL‐17, IFN‐γ, TGF‐β

Abbreviations: *B*., Bifidobacterium; CFU, colony‐forming unit; CRP, C‐reactive protein; DBP, diastolic blood pressure; *E*., enterococcus; FPG, fasting plasma glucose; HbA1c, glycosylated hemoglobin; HDL, high‐density lipoprotein; *hs*‐CRP, high‐sensitivity C‐reactive protein; IFN‐γ, interferon‐gamma; IL‐10, interleukin‐10; IL‐6, interleukin‐6; *L*., lactobacillus; LDL, low‐density lipoprotein; NI, no information; *S*., streptococcus; SBP, systolic blood pressure; TC, total cholesterol; TG, triglyceride; TGF‐β, transforming growth factor‐beta; TNF‐α, t*umor necrosis factor*‐alpha.

### 3.2 Risk of Bias With Individual Studies

**FIGURE 2:**
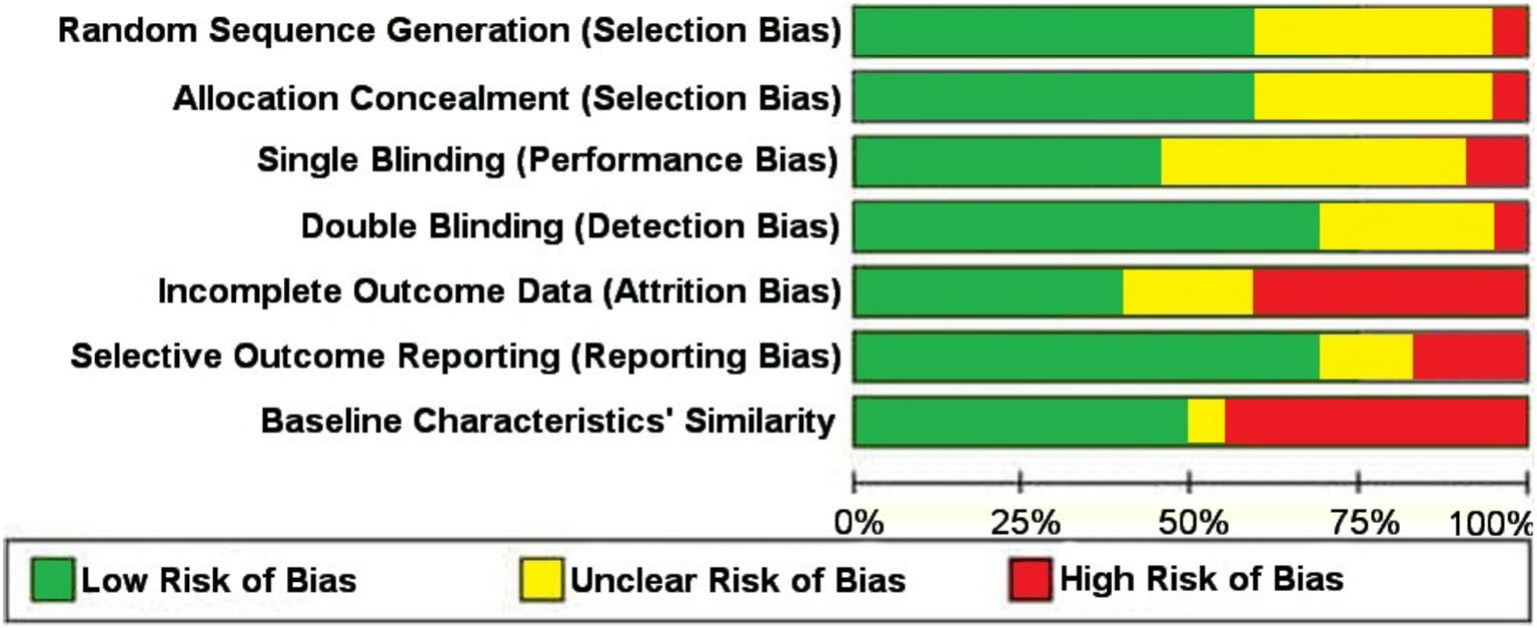
Risk of bias graph for included studies.

The Cochrane Collaboration tool for the assessment of RCTs was used to assess the risk of bias within individual trials (Figure 2 and Table S2). Six trials showed a low overall risk of bias as they presented it in the main domains (Choi et al. 2022; Toshimitsu et al. 2021; Naito et al. 2017; Schellekens et al. 2021; Zarrati et al. 2014, 2013). Ten trials did not describe the sequence generation and allocation concealment adequately (Orak et al. 2022; Ben Othman et al. 2023; Cho et al. 2022; Sohn, Na, et al. 2022; Agerholm‐Larsen et al. 2000; Krumbeck et al. 2018; Lim et al. 2020; Déchelotte et al. 2021; Rahayu et al. 2021; Rajkumar et al. 2014). Thirteen trials were non‐blinded or did not clearly present the method for blinding participants, healthcare providers, and/or outcome assessors (Madjd et al. 2016; Orak et al. 2022; Ben Othman et al. 2023; Sohn, Jung, et al. 2022; Cho et al. 2022; Sohn, Na, et al. 2022; Agerholm‐Larsen et al. 2000; Gomes et al. 2017; Krumbeck et al. 2018; Lim et al. 2020; Déchelotte et al. 2021; Rajkumar et al. 2014; Razmpoosh et al. 2020). In 15 RCTs, incomplete outcome data was observed owing to post‐randomization inclusions (Stenman et al. 2016; Hajipoor et al. 2022, 2021; Sohn, Jung, et al. 2022; Cho et al. 2022; de Oliveira 2020; Agerholm‐Larsen et al. 2000; Gomes et al. 2017; Jung et al. 2015; Krumbeck et al. 2018; Lim et al. 2020; Nasiri et al. 2020; Rahayu et al. 2021; Rajkumar et al. 2014; Razmpoosh et al. 2020). Moreover, 9 studies showed a high or unclear risk of bias in selective outcome reporting (Orak et al. 2022; Ben Othman et al. 2023; Hajipoor et al. 2022, 2021; Gomes et al. 2017; Naito et al. 2017; Zarrati et al. 2014, 2013; Razmpoosh et al. 2020). Finally, there was a high or unclear risk of bias on baseline similarities of the intervention and control groups in 13 trials (Orak et al. 2022; Hajipoor et al. 2022, 2021; Toshimitsu et al. 2021; de Oliveira 2020; Agerholm‐Larsen et al. 2000; Gomes et al. 2017; Jung et al. 2015; Krumbeck et al. 2018; Naito et al. 2017; Rahayu et al. 2021; Rajkumar et al. 2014; Razmpoosh et al. 2020).

### 3.3 Findings From the Meta‐Analysis and Subgroup Analysis

#### 3.3.1 Effects on Systolic and Diastolic Blood Pressure

**FIGURE 3:**
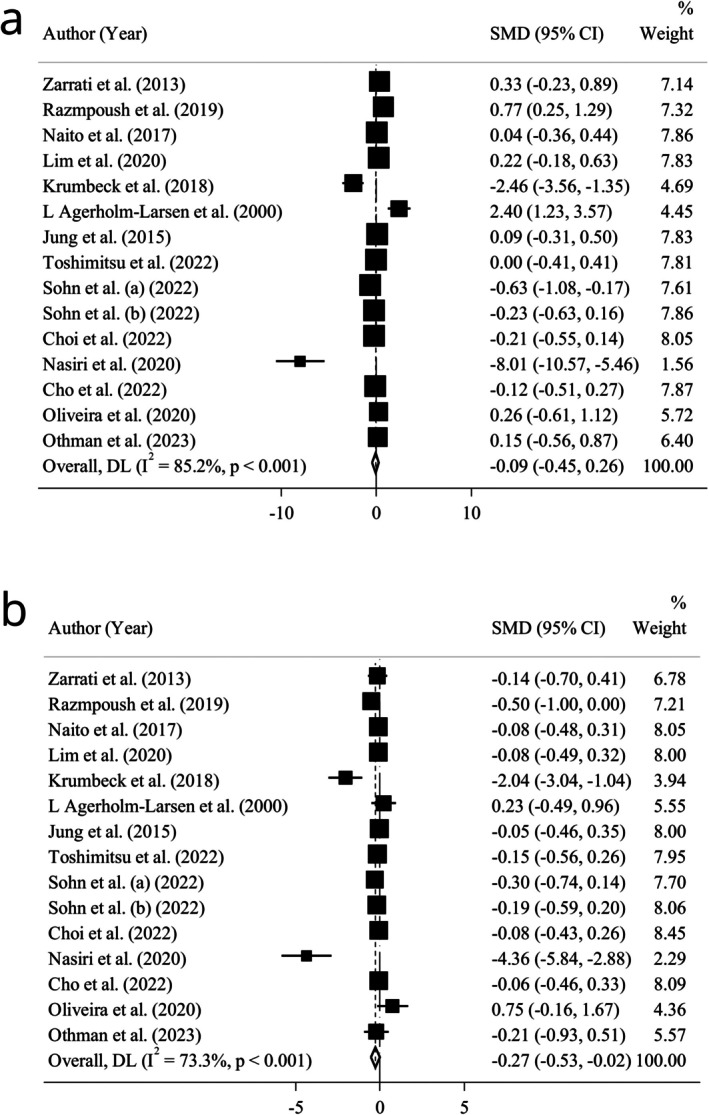
Forest plot of the effect of probiotics on (a) systolic and (b) diastolic blood pressure.

There were 15 RCTs, including 1045 individuals, which presented the pooled effect of probiotic intake on SBP and DBP. A trivial and not statistically significant efficacy was observed for SBP reduction (*n* = 15; SMD = −0.09; 95% CI: −0.45, 0.26; *p* = 0.605) (Figure 3). Heterogeneity test results also showed a highly severe heterogeneity (*I* ^2^ = 85.2%; *p* = 0.000). We conducted subgroup analysis for SBP and DBP based on age, gender, BMI, health condition, delivery format, as well as supplementation type, dosage, and duration (Table 2 and Figure S1). Although subgroup analysis according to health status and supplementation dosage decreased statistical heterogeneity in healthy individuals and in adults who received a supplementation dosage of < 10^9^ cfu per day, an incremental impact on SBP was observed in these groups after receiving probiotics. Moreover, there was a low‐sized and medium‐sized increasing effect on SBP in individuals with a treatment duration of eight weeks or less, as well as in participants consuming probiotic‐fermented dairy products, respectively. In contrast, subgroup analysis according to the duration of the supplementation and forms of administration demonstrated a low‐sized SBP‐lowering effect of probiotics in participants supplemented for more than eight weeks and in individuals who used probiotic tablets or capsules instead of fermented products and sachets.

**TABLE 2:** Subgroup analysis of systolic and diastolic blood pressure.

Subgroup by	*p* for heterogeneity between subgroups (test for interaction)	No. of trials	No. of participants	SMD	95% CI	*I* ^2^ (%)	*p* for heterogeneity
*Changes in SBP*							
Age	0.143						
< 42		8	534	0.03	−0.59, 0.66	89.4	0
≥ 42		7	511	−0.24	−0.62, 0.14	77.0	0
Gender	0.008						
Male		1	98	0.04	−0.36, 0.44	NA	NA
Female		2	86	0.63	0.19, 1.08	0	0.320
Both		12	861	−0.22	−0.64, 0.19	86.9	0
BMI	0.099						
25 ≤ BMI < 30		8	686	−0.27	−0.69, 0.15	84.9	0
≥ 30		7	359	0.18	−0.50, 0.86	86.8	0
Health status	0.082						
Healthy		4	342	0.21	−0.20, 0.62	69.7	0.019
With comorbidities		11	703	−0.27	−0.76, 0.21	87.7	0
Supplementation type	0.008						
Single‐type probiotics		8	658	−0.09	−0.56, 0.09	73.7	0
Multiple‐type probiotics		7	387	0.03	−0.72, 0.78	90.1	0
Supplementation dosage	0.004						
< 10^9^ cfu		2	115	0.56	0.13, 0.99	21.8	0.258
10^9^ cfu ≤ Dose < 10^10^ cfu		6	320	−0.12	−0.82, 0.58	87.6	0
≥ 10^10^ cfu		7	610	−0.23	−0.72, 0.25	85.6	0
Duration of intervention	0.009						
≤ 8 weeks		6	304	0.21	−0.54, 0.97	87.8	0
> 8 weeks		9	741	−0.24	−0.63, 0.14	82.9	0
Delivery format	0.003						
Dairy products		5	335	0.52	−0.01, 1.04	79.2	0.001
Sachets or powder		2	126	−1.13	−3.62, 1.37	94.4	0
Capsules or tablets		8	584	−0.32	−0.79, 0.15	84.6	0
Quality of trials	0.992						
High quality		4	372	−0.02	−0.22, 0.19	0	0.443
Moderate quality		3	158	−1.98	−4.35, 0.39	94.8	0
Low quality		8	515	0	−0.53, 0.53	86.9	0
*Changes in DBP*							
Age	0.849						
< 42		8	534	−0.30	−0.74, 0.15	81.6	0
≥ 42		7	511	−0.26	−0.54, 0.02	58.2	0.026
Gender	0.880						
Male		1	98	−0.08	−0.48, 0.31	NA	NA
Female		2	86	0.07	−1.16, 1.29	81.9	0.019
Both		12	861	−0.34	−0.64, −0.04	76.5	0
BMI	0.862						
25 ≤ BMI < 30		8	686	−0.30	−0.64, 0.04	78.2	0
≥ 30		7	359	−0.24	−0.67, 0.19	70.6	0.002
Health status	0.808						
Healthy		4	342	−0.16	−0.37, 0.06	0	0.523
With comorbidities		11	703	−0.35	−0.72, 0.01	80.1	0
Supplementation type	0.688						
Single‐type probiotics		8	658	−0.19	−0.46, 0.08	61.7	0.011
Multiple‐type probiotics		7	387	−0.44	−0.96, 0.08	82.4	0
Supplementation dosage	0.672						
< 10^9^ cfu		2	115	−0.34	−0.71, 0.03	0	0.350
10^9^ cfu ≤ Dose < 10^10^ cfu		6	320	−0.20	−0.66, 0.25	73.9	0.002
≥ 10^10^ cfu		7	610	−0.35	−0.76, 0.06	81.1	0
Duration of intervention	0.365						
≤ 8 weeks		6	304	−0.36	−0.80, 0.08	68.3	0.008
> 8 weeks		9	741	−0.23	−0.56, 0.10	77.7	0
Delivery format	0.705						
Dairy products		5	335	−0.16	−0.38, 0.06	0	0.549
Sachets or powder		2	126	−0.99	−2.94, 0.95	92.3	0
Capsules or tablets		8	584	−0.29	−0.70, 0.12	80.4	0
Quality of trials	0.651						
High quality		4	372	−0.02	−0.22, 0.19	0	0.443
Moderate quality		3	158	−1.98	−4.35, 0.39	94.8	0
Low quality		8	515	0	−0.53, 0.53	86.9	0

Abbreviations: BMI, body mass index; cfu, colony‐forming unit; CI, confidence interval; DBP, diastolic blood pressure; NA, not applicable; SBP, systolic blood pressure; SMD, standardized mean difference.

Data pooling revealed a significantly low‐sized efficacy in reducing DBP (*n* = 15; SMD = −0.27; 95% CI: −0.53, −0.02; *p* = 0.037) (Figure 3). Besides, heterogeneity test results showed severe statistical heterogeneity (*I* ^2^ = 73.3%; *p* = 0.000). However, the results of the subgroup analysis based on health status, supplementation dosage, and delivery format could result in a considerable reduction in statistical heterogeneity in healthy people, as well as those with supplementation dosages < 10^9^ cfu, and in participants who consumed probiotic‐fermented dairy products, respectively (Table 2 and Figures S9–S16). Also, there was a low‐sized decreasing impact on DBP in people with comorbidities, participants receiving multiple types of probiotics, and those who used a supplementation dosage of 10^10^ cfu or more. Furthermore, a large‐sized lowering impact on DBP was observed in people who consumed probiotic sachets or powder instead of fermented products, capsules, and tablets.

There was no relationship between the quality of trials and meta‐analysis results related to SBP and DBP after subgroup analysis, based on the quality of studies (Table 2).

#### 3.3.2 Effects on Fasting Plasma Glucose, Hemoglobin A1c, and Fasting Insulin

**FIGURE 4:**
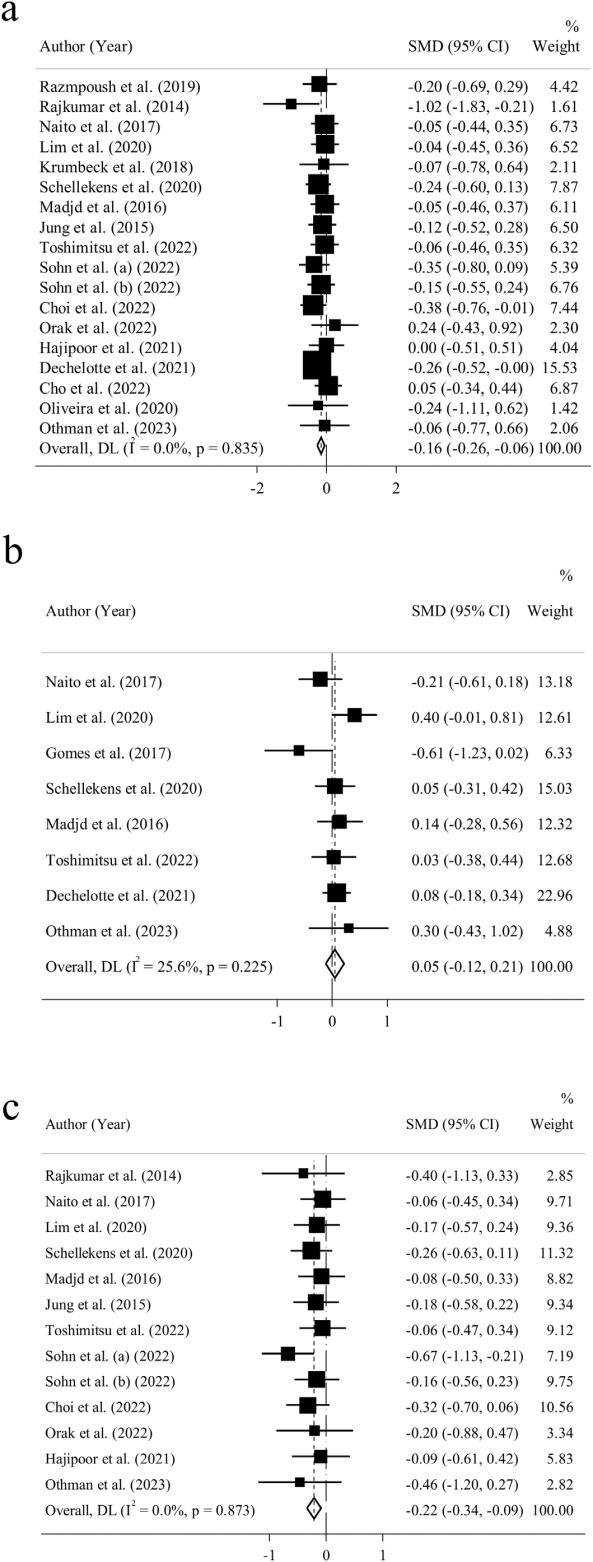
Forest plot of the effect of probiotics on (a) fasting plasma glucose, (b) glycosylated hemoglobin, (c) fasting insulin.

There were 18 RCTs including 1469 adults that presented the pooled effect of probiotic administration on FPG. A trivial, but statistically significant efficacy was observed in the reduction of FPG (*n* = 18; SMD = −0.16; 95% CI: −0.26, 0.06; *p* = 0.002) along with a mild statistical heterogeneity (*I* ^2^ = 0%; *p* = 0.835) (Figure 4). Subgroup analysis for glycemic indices was executed according to age, gender, BMI, health condition, delivery format, as well as supplementation type, dosage, and duration (Table 3 and Figure S17–S24). The results of the subgroup analysis illustrated a low‐sized reducing impact on FPG in healthy participants, trials with supplementation dosages of 10^10^ cfu or more, and adults who used capsules or tablets as their form of administration.

**TABLE 3:** Subgroup analysis of glycemic indices.

Subgroup by	*p* for heterogeneity between subgroups (test for interaction)	No. of trials	No. of participants	SMD	95% CI	*I* ^2^ (%)	*p* for heterogeneity
*Changes of FPG*							
Age	0.744						
< 42		8	594	−0.14	−0.31, 0.02	0	0.843
≥ 42		8	633	−0.11	−0.26, 0.05	0	0.924
Gender	0.620						
Male		1	98	−0.05	−0.44, 0.35	NA	NA
Female		4	209	−0.07	−0.34, 0.20	0	0.738
Both		13	1162	−0.19	−0.30, −0.07	0	0.687
BMI	0.871						
25 ≤ BMI < 30		8	856	−0.14	−0.27, −0.01	0	0.860
≥ 30		9	583	−0.16	−0.32, 0.01	0	0.881
Health status	0.443						
Healthy		7	567	−0.21	−0.40, −0.02	16.2	0.307
With comorbidities		11	902	−0.13	−0.26, 0	0	0.962
Supplementation type	0.294						
Single‐type probiotics		10	992	−0.20	−0.33, −0.07	0	0.943
Multiple‐type probiotics		8	477	−0.08	−0.26, 0.09	0	0.445
Supplementation dosage	0.458						
< 10^9^ cfu		3	213	−0.08	−0.35, 0.19	0	0.841
10^9^ cfu ≤ Dose < 10^10^ cfu		6	324	−0.08	−0.28, 0.13	0	0.714
≥ 10^10^ cfu		9	932	−0.21	−0.34, −0.08	0	0.583
Duration of intervention	0.797						
≤ 8 weeks		6	288	−0.14	−0.41, 0.13	18.3	0.295
> 8 weeks		12	1181	−0.17	−0.28, −0.05	0	0.920
Delivery format	0.477						
Dairy products		5	403	−0.07	−0.26, 0.13	0	0.985
Sachets or powder		2	126	−0.11	−0.46, 0.24	0	0.909
Capsules or tablets		11	940	−0.21	−0.34, −0.08	0	0.482
Quality of trials	0.871						
High quality		4	444	−0.19	−0.38, 0	0	0.577
Moderate quality		4	264	−0.10	−0.38, 0.17	0	0.917
Low quality		10	761	−0.17	−0.31, −0.03	0	0.477
*Changes of HbA1c*							
Age	0.408						
< 42		2	132	−0.20	−0.92, 0.53	73.6	0.052
≥ 42		5	456	0.08	−0.13, 0.29	17.8	0.302
Gender	0.203						
Male		1	98	−0.21	−0.61, 0.18	NA	NA
Female		2	132	−0.20	−0.92, 0.53	73.6	0.052
Both		5	570	0.13	−0.04, 0.30	0	0.657
BMI	0.690						
25 ≤ BMI < 30		4	516	0.07	−0.15, 0.29	33.2	0.213
≥ 30		4	284	−0.01	−0.32, 0.31	36.9	0.191
Health status	0.491						
Healthy		3	254	−0.07	−0.44, 0.31	51.1	0.129
With comorbidities		5	546	0.09	−0.11, 0.28	17.3	0.304
Supplementation type	0.632						
Single‐type probiotics		5	638	0.07	−0.10, 0.24	11.1	0.343
Multiple‐type probiotics		3	162	−0.05	−0.56, 0.46	57.2	0.097
Supplementation dosage	0.904						
< 10^9^ cfu		1	89	0.14	−0.28, 0.56	NA	NA
10^9^ cfu ≤ Dose < 10^10^ cfu		1	92	0.03	−0.38, 0.44	NA	NA
≥ 10^10^ cfu		6	619	0.03	−0.21, 0.26	45.7	0.101
Duration of intervention	0.052						
≤ 8 weeks		3	171	−0.21	−0.63, 0.22	41.5	0.181
> 8 weeks		5	629	0.12	−0.03, 0.28	0	0.696
Delivery format	0.057						
Dairy products		3	279	−0.02	−0.26, 0.21	0	0.465
Sachets or powder		1	43	−0.61	−1.23, 0.02	NA	NA
Capsules or tablets		4	478	0.15	−0.03, 0.33	0	0.543
Quality of trials	0.589						
High quality		3	312	−0.04	−0.26, 0.18	0	0.581
Moderate quality		1	89	0.14	−0.28, 0.56	NA	NA
Low quality		4	399	0.07	−0.29, 0.43	58.7	0.064
*Changes of fasting insulin*							
Age	0.659						
< 42		6	508	−0.18	−0.36, 0	0	0.972
≥ 42		6	527	−0.24	−0.42, −0.06	7.9	0.366
Gender	0.557						
Male		1	98	−0.06	−0.45, 0.34	NA	NA
Female		2	123	−0.12	−0.47, 0.24	0	767
Both		10	844	−0.25	−0.39, −0.11	0	0.789
BMI	0.836						
25 ≤ BMI < 30		6	569	−0.20	−0.37, −0.03	1.1	0.409
≥ 30		6	466	−0.23	−0.41, −0.04	0	0.925
Health status	0.845						
Healthy		6	502	−0.23	−0.41, −0.05	0	0.961
With comorbidities		7	563	−0.20	−0.37, −0.04	0	0.458
Supplementation type	0.727						
Single‐type probiotics		7	728	−0.23	−0.38, −0.08	0	0.496
Multiple‐type probiotics		6	337	−0.18	−0.40, 0.03	0	0.940
Supplementation dosage	0.590						
< 10^9^ cfu		2	148	−0.09	−0.41, 0.24	0	0.975
10^9^ cfu ≤ Dose < 10^10^ cfu		3	197	−0.32	−0.72, 0.08	48.4	0.144
≥ 10^10^ cfu		8	720	−0.22	−0.37, −0.06	0	0.969
Duration of intervention	0.885						
≤ 8 weeks		4	192	−0.20	−0.48, 0.09	0	0.731
> 8 weeks		9	873	−0.22	−0.36, −0.08	0	0.709
Delivery format	0.230						
Dairy products		4	338	−0.07	−0.29, 0.14	0	0.999
Sachets or powder		1	95	−0.18	−0.58, 0.22	NA	NA
Capsules or tablets		8	632	−0.31	−0.47, −0.14	0	0.803
Quality of trials	0.524						
High quality		4	444	−0.18	−0.38, 0.01	0	0.704
Moderate quality		3	243	−0.13	−0.42, 0.16	0	0.745
Low quality		6	378	−0.32	−0.52, −0.11	0	0.590

Abbreviations: BMI, body mass index; cfu, colony‐forming unit; CI, confidence interval; FPG, fasting plasma glucose; HbA1c, glycosylated hemoglobin; NA, not applicable; SMD, standardized mean difference.

Eight trials including 800 individuals presented the pooled effect of probiotic intake on glycosylated hemoglobin (HbA1c). A trivial nonsignificant incremental effect of probiotics was observed on the HbA1c level (*n* = 8; SMD = 0.05; 95% CI: −0.12, 0.21; *p* = 0.475) (Figure 4). We also observed a moderate statistical heterogeneity after performing the heterogeneity test (*I* ^2^ = 25.6%; *p* = 0.225). Subgroup analyses reduced statistical heterogeneity in 42‐year‐old individuals and older, people with comorbidities, participants who used single‐type probiotics, trials that lasted for more than 8 weeks, and adults who consumed probiotic capsules or tablets instead of fermented products and sachets (Table 3 and Figures S25–S32). Nevertheless, there was still a trivial incremental effect of probiotics on HbA1c levels in these groups. In contrast, a trivial lowering impact of probiotics on HbA1c level was observed in adults who used probiotic‐fermented dairy products, people younger than 42, obese participants, healthy individuals, and those who consumed multiple types of probiotics.

There were 13 trials including 1065 participants that indicated the pooled effect of probiotics on fasting insulin. Data pooling showed that probiotic treatment could lead to a low‐sized, but yet significant decrease in fasting insulin (*n* = 13; SMD = −0.22; 95% CI: −0.34, −0.09; *p* = 0.001) (Figure 4) along with a mild statistical heterogeneity (*I* ^2^ = 0%; *p* = 0.873). Moreover, subgroup analysis according to the supplementation dosage and delivery format revealed a mild increase in the effectiveness of probiotics in reducing fasting insulin in individuals receiving a supplementation dosage of 10^9^ cfu ≤ Dose < 10^10^ cfu and those who consumed probiotic capsules or tablets instead of probiotic‐fermented dairy products and sachets (Table 3 and Figures S33–S40).

There was no relationship between the quality of studies and meta‐analysis results related to FPG, HbA1c, and fasting serum insulin after subgroup analysis based on the quality of studies (Table 3).

#### 3.3.3 Effects on Lipid Profile

**FIGURE 5:**
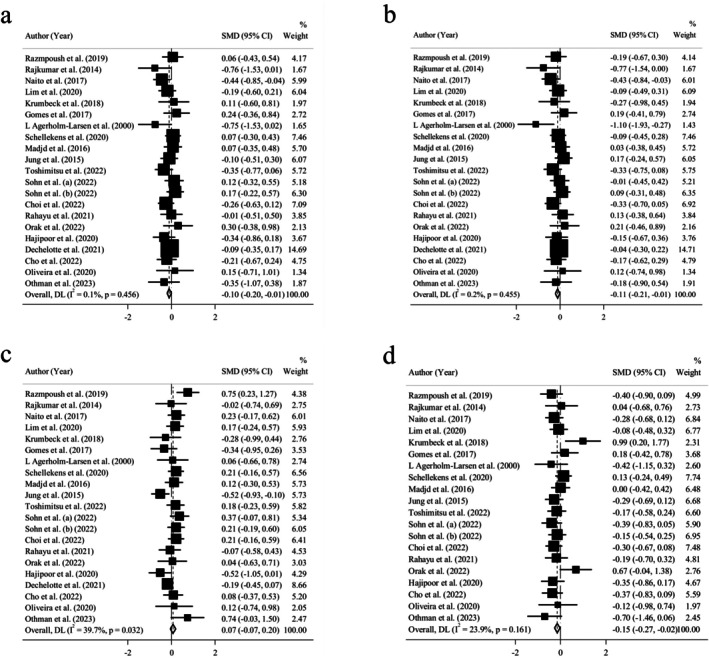
Forest plot of the effect of probiotics on (a) total cholesterol, (b) low‐density lipoprotein, (c) high‐density lipoprotein, and (d) triglyceride.

Twenty‐one trials including 1602 participants presented the pooled effect of probiotic administration on TC, LDL‐C, and HDL‐C. A trivial yet significant efficacy was observed in the reduction of TC level (*n* = 21; SMD = −0.10; 95% CI: −0.20, −0.01; *p* = 0.039) (Figure 5). In spite of mild heterogeneity (*I* ^2^ = 0.1%; *p* = 0.456) for TC, subgroup analysis was executed based on age, gender, forms of administration, BMI, health status, supplementation type, dosage, and duration for all lipid profile parameters, including TC, LDL‐C, HDL‐C, and triglyceride (Table 4 and Figures S41–S48). A low‐sized lowering impact of probiotics was observed on the TC concentrations in trials that used probiotic‐fermented dairy products as their delivery format. Meanwhile, there was a mild increase in the efficacy of supplementation to reduce serum TC in adults with comorbidities and those who were supplemented for 8 weeks or less. In contrast, a trivial incremental effect of the intervention on TC concentration was revealed in women and those who consumed sachets or powder instead of fermented products, tablets, and capsules.

**TABLE 4:** Subgroup analysis of lipid profile.

Subgroup by	*p* for heterogeneity between subgroups (test for interaction)	No. of trials	No. of participants	SMD	95% CI	*I* ^2^ (%)	*p* for heterogeneity
*Changes in TC*							
Age	0.358						
< 42		10	667	−0.04	−0.20, 0.12	4.6	0.398
≥ 42		9	693	−0.14	−0.30, 0.01	0	0.547
Gender	0.044						
Male		1	98	−0.44	−0.85, −0.04	NA	NA
Female		5	252	0.13	−0.12, 0.38	0	0.968
Both		15	1252	−0.13	−0.24, −0.02	0	0.511
BMI	0.440						
25 ≤ BMI < 30		8	856	−0.13	−0.27, 0.01	2.5	0.411
≥ 30		12	716	−0.05	−0.20, 0.10	0	0.584
Health status	0.234						
Healthy		9	670	−0.03	−0.19, 0.12	0	0.507
With comorbidities		12	932	−0.16	−0.29, −0.02	2.9	0.456
Supplementation type	0.821						
Single‐type probiotics		11	1052	−0.10	−0.22, 0.03	0	0.496
Multiple‐type probiotics		10	550	−0.13	−0.31, 0.06	15.0	0.305
Supplementation dosage	0.891						
< 10^9^ cfu		3	213	−0.05	−0.32, 0.22	0	0.430
10^9^ cfu ≤ Dose < 10^10^ cfu		8	414	−0.10	−0.30, 0.10	6.2	0.382
≥ 10^10^ cfu		10	975	−0.12	−0.27, 0.02	15.4	0.301
Duration of intervention	0.406						
≤ 8 weeks		8	361	−0.18	−0.46, 0.10	38.5	0.122
> 8 weeks		13	1241	−0.08	−0.19, 0.03	0	0.790
Delivery format	0.215						
Dairy products		6	433	−0.25	−0.47, −0.03	23.6	0.257
Sachets or powder		4	229	0.01	−0.25, 0.27	0	0.819
Capsules or tablets		11	970	−0.07	−0.20, 0.06	0	0.488
Quality of trials	0.352						
High quality		4	444	−0.23	−0.45, −0.01	24.3	0.265
Moderate quality		4	264	−0.08	−0.32, 0.17	0	0.628
Low quality		13	894	−0.06	−0.19, 0.08	1.9	0.427
*Changes of LDL*							
Age	0.339						
< 42		10	667	−0.06	−0.24, 0.12	22.8	0.233
≥ 42		9	693	−0.16	−0.31, −0.01	0	0.817
Gender	0.154						
Male		1	98	−0.43	−0.84, −0.03	NA	NA
Female		5	252	0.03	−0.21, 0.28	0	0.856
Both		15	1252	−0.12	−0.24, 0	6.5	0.380
BMI	0.837						
25 ≤ BMI < 30		8	856	−0.09	−0.23, 0.04	0	0.458
≥ 30		12	716	−0.11	−0.26, 0.04	0	0.491
Health status	0.366						
Healthy		9	670	−0.06	−0.22, 0.11	7.1	0.376
With comorbidities		12	932	−0.15	−0.28, −0.02	0	0.476
Supplementation type	0.785						
Single‐type probiotics		11	1052	−0.12	−0.24, 0	0	0.678
Multiple‐type probiotics		10	550	−0.11	−0.32, 0.09	27.8	0.188
Supplementation dosage	0.928						
< 10^9^ cfu		3	213	−0.08	−0.35, 0.18	0	0.762
10^9^ cfu ≤ Dose < 10^10^ cfu		8	414	−0.14	−0.37, 0.08	21.1	0.262
≥ 10^10^ cfu		10	975	−0.11	−0.25, 0.03	14.1	0.313
Duration of intervention	0.083						
≤ 8 weeks		8	361	−0.28	−0.55, −0.01	32.5	0.168
> 8 weeks		13	1241	−0.07	−0.18, 0.05	0	0.880
Delivery format	0.071						
Dairy products		6	433	−0.28	−0.51, −0.05	27.3	0.230
Sachets or powder		4	229	0.10	−0.16, 0.36	0	0.749
Capsules or tablets		11	970	−0.09	−0.22, 0.04	0	0.756
Quality of trials	0.087						
High quality		4	444	−0.28	−0.48, −0.09	0	0.630
Moderate quality		4	264	0.05	−0.20, 0.29	0	0.813
Low quality		13	894	−0.08	−0.22, 0.05	3.9	0.407
*Changes of HDL*							
Age	0.159						
< 42		10	667	0.01	−0.24, 0.27	58.2	0.010
≥ 42		9	693	0.18	0.03, 0.33	0	0.700
Gender	0.376						
Male		1	98	0.23	−0.17, 0.62	NA	NA
Female		5	252	0.16	−0.21, 0.52	48.5	0.101
Both		15	1252	0.03	−0.12, 0.18	40.3	0.053
BMI	0.444						
25 ≤ BMI < 30		8	856	0.05	−0.15, 0.25	51.1	0.046
≥ 30		12	716	0.09	−0.11, 0.29	39.7	0.076
Health status	0.998						
Healthy		9	670	0.05	−0.19, 0.29	54.3	0.025
With comorbidities		12	932	0.08	−0.08, 0.24	29.7	0.154
Supplementation type	0.303						
Single‐type probiotics		11	1052	0.10	−0.03, 0.22	0	0.510
Multiple‐type probiotics		10	550	0.01	−0.27, 0.29	60.7	0.006
Supplementation dosage	0.690						
< 10^9^ cfu		3	213	0.12	−0.55, 0.78	82.2	0.004
10^9^ cfu ≤ Dose < 10^10^ cfu		8	414	0.11	−0.09, 0.30	0	0.872
≥ 10^10^ cfu		10	975	0.04	−0.15, 0.24	50.1	0.035
Duration of intervention	0.183						
≤ 8 weeks		8	361	0.16	−0.12, 0.45	40.4	0.110
> 8 weeks		13	1241	0.03	−0.12, 0.18	39	0.074
Delivery format	0.007						
Dairy products		6	433	0.15	−0.15, 0.44	56.6	0.042
Sachets or powder		4	229	−0.33	−0.60, −0.07	0	0.616
Capsules or tablets		11	970	0.11	−0.02, 0.24	0	0.443
Quality of trials	0.018						
High quality		4	444	0.21	0.01, 0.40	0	0.999
Moderate quality		4	264	−0.24	−0.61, 0.14	52.4	0.098
Low quality		13	894	0.10	−0.07, 0.28	36.1	0.094
*Changes of TG*							
Age	0.872						
< 42		10	667	−0.16	−0.33, 0	8.6	0.363
≥ 42		9	693	−0.15	0.36, 0.07	46.0	0.063
Gender	0.364						
Male		1	98	−0.28	−0.68, 0.12	NA	NA
Female		5	252	0.03	−0.30, 0.36	37.3	0.172
Both		14	1040	−0.18	−0.32, −0.03	21.6	0.219
BMI	0.158						
25 ≤ BMI < 30		7	644	−0.24	−0.39, −0.08	0	0.942
≥ 30		12	716	−0.07	−0.29, 0.15	47.5	0.034
Health status	0.216						
Healthy		9	670	−0.07	−0.26, 0.11	24.8	0.223
With comorbidities		11	720	−0.22	−0.39, −0.04	22.0	0.234
Supplementation type	0.607						
Single‐type probiotics		10	840	−0.12	−0.29, 0.05	27.8	0.188
Multiple‐type probiotics		10	550	−0.18	−0.38, 0.02	26.5	0.200
Supplementation dosage	0.878						
< 10^9^ cfu		3	213	−0.22	−0.49, 0.06	0	0.402
10^9^ cfu ≤ Dose < 10^10^ cfu		8	414	−0.06	−0.37, 0.24	55.2	0.029
≥ 10^10^ cfu		9	763	−0.15	−0.29, 0	0	0.509
Duration of intervention	0.462						
≤ 8 weeks		8	361	−0.02	−0.38, 0.34	61.4	0.011
> 8 weeks		12	1029	−0.18	−0.30, −0.05	0	0.853
Delivery format	0.448						
Dairy products		6	433	−0.23	−0.42, −0.04	0	0.819
Sachets or powder		4	229	0.09	−0.38, 0.57	66.0	0.032
Capsules or tablets		10	728	−0.14	−0.32, 0.04	27.1	0.195
Quality of trials	0.948						
High quality		4	444	−0.15	−0.35, 0.05	8.1	0.353
Moderate quality		4	264	−0.19	−0.43, 0.05	0	0.706
Low quality		12	682	−0.11	−0.33, 0.10	45.6	0.043

Abbreviations: BMI, body mass index; cfu, colony‐forming unit; CI, confidence interval; HDL, high‐density lipoprotein; LDL, low‐density lipoprotein; NA, not applicable; SMD, standardized mean difference; TC, total cholesterol; TG, triglyceride.

Data pooling demonstrated a trivial and significant efficacy in LDL‐C reduction (*n* = 21; SMD = −0.11; 95% CI: −0.21, −0.01; *p* = 0.020) (Figure 5). In addition, heterogeneity test results indicated a mild statistical heterogeneity (*I* ^2^ = 0.2%; *p* = 0.455). Although subgroup analysis reported a low‐sized decreasing effect of supplementation on LDL‐C level in trials that chose probiotic‐fermented dairy products as their form of administration and individuals supplemented for 8 weeks or less, we observed a trivial but increasing impact on serum LDL‐C in women and participants intaking probiotic sachets or powder instead of dairy products, capsules, and tablets (Table 4 and Figures S49–S56).

Our meta‐analysis revealed a nonsignificant trivial efficacy in HDL‐C rise (*n* = 21; SMD = 0.07; 95% CI: −0.07, 0.20; *p* = 0.316) (Figure 5). In addition, a moderate statistical heterogeneity was observed, based on the heterogeneity test results (*I* ^2^ = 39.7%; *p* = 0.032). However, subgroup analysis removed the heterogeneity in 42‐year‐old participants or older, trials with single‐type probiotics, trials with supplementation dosages of 10^9^ cfu ≤ Dose < 10^10^ cfu, and participants who used probiotic capsules, tablets, or sachets instead of probiotic‐fermented dairy products (Table 4 and Figures S57–S64). Moreover, a mild rise was observed in the efficacy of the intervention in 42‐year‐old participants or older, female participants, individuals who used probiotics for 8 weeks or less, and studies that used probiotic‐fermented dairy products as their delivery format. In contrast, there was a low‐sized incremental effect of probiotic supplementation on HDL‐C concentration in people consuming probiotic sachets instead of probiotic capsules, tablets, and dairy products.

Twenty trials including 1390 participants presented the pooled effect of probiotic intake on triglyceride (TG). The meta‐analysis results demonstrated significant trivial efficacy in TG reduction (*n* = 20; SMD = −0.15; 95% CI: −0.27, −0.02; *p* = 0.022), accompanied by a mild statistical heterogeneity (*I* ^2^ = 23.9%; *p* = 0.161) (Figure 5). However, subgroup analysis ruled out the heterogeneity in overweight non‐obese adults, participants consuming supplementation dosages < 10^9^ cfu or more than 10^10^ cfu, trials that lasted for more than 8 weeks, and individuals who used probiotic‐fermented dairy products instead of probiotic sachets, capsules, and tablets (Table 4 and Figures S65–S72). Additionally, subgroup analysis illustrated a small‐sized reducing impact of the intervention on TG concentration in overweight non‐obese adults, individuals with comorbidities, studies with intervention dosages < 10^9^ cfu, and participants who used probiotic‐fermented dairy products. In contrast, there was a trivial incremental impact on TG concentrations in female participants and trials that chose probiotic sachets as their delivery format.

Subgroup analysis according to the quality of trials revealed no relationship between the quality of studies and meta‐analysis results related to lipid profile parameters (Table 4).

#### 3.3.4 Effects on CRP Level

**FIGURE 6:**
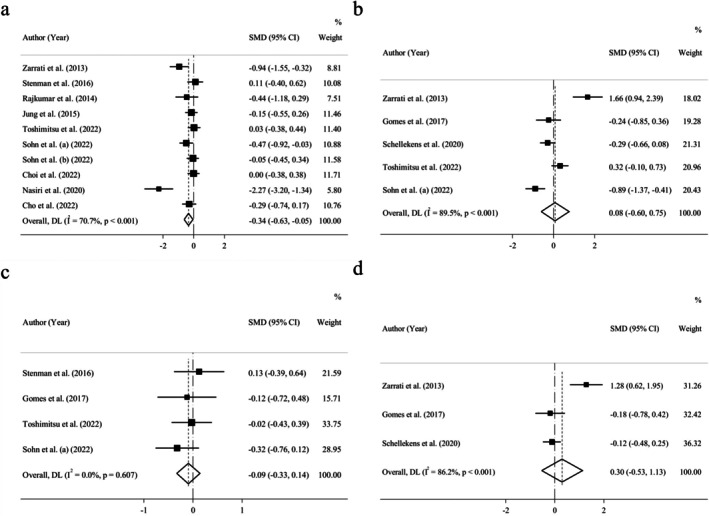
Forrest plot of the effect of probiotics on (a) C‐reactive protein, (b) tumor necrosis factor‐alpha, (c) interleukin‐6, and (d) interleukin‐10.

Ten RCTs including 747 participants showed the pooled effect of probiotics on CRP concentration. There was a significant low‐sized lowering impact on CRP level (*n* = 10; SMD = −0.34; 95% CI: −0.63, −0.05; *p* = 0.020) (Figure 6). Heterogeneity test results presented severe statistical heterogeneity (*I* ^2^ = 70.7%; *p* = 0.000). Subgroup analysis for CRP was executed according to age, gender, BMI, health condition, delivery format, as well as supplementation type, dosage, and duration. Subgroup analysis could reduce the statistical heterogeneity in 42‐year‐old participants and older, healthy individuals, adults who used single‐type probiotics, trials with supplementation dosages of 10^9^ cfu ≤ Dose < 10^10^ cfu, individuals supplemented for 8 weeks or less, and those who consumed probiotic sachets instead of capsules, tablets, and dairy products (Table 5 and Figures S73–S80). Moreover, we observed a medium‐sized decreasing effect of the intervention on CRP levels in people younger than 42, those who used multiple‐type probiotics, and participants who consumed probiotics for 8 weeks or less. Subgroup analysis also revealed a mild improvement in the efficacy of the intervention to reduce CRP levels in overweight people, participants with comorbidities, and those who used capsules, tablets, or probiotic‐fermented dairy products instead of probiotic sachets. No relationship was observed between the quality of studies and meta‐analysis results related to CRP concentration, after subgroup analysis according to the quality of trials (Table 5).

**TABLE 5:** Subgroup analysis of C‐reactive protein.

Subgroup by	p for heterogeneity between subgroups (test for interaction)	No. of trials	No. of participants	SMD	95% CI	*I* ^2^ (%)	*p* for heterogeneity
*Changes of CRP*							
Age	0.467						
< 42		5	418	−0.56	−1.11, 0	84.5	0
≥ 42		4	299	−0.16	−0.42, 0.10	25.8	0.257
Gender	0.051						
Male		0	NA	NA	NA	NA	NA
Female		0	NA	NA	NA	NA	NA
Both		10	747	−0.34	−0.63, −0.05	70.7	0
BMI	0.567						
25 ≤ BMI < 30		6	474	−0.40	−0.80, 0	77.4	0
≥ 30		3	243	−0.24	−0.81, 0.33	74.6	0.020
Health status	0.507						
Healthy		5	368	−0.23	−0.56, 0.10	52.5	0.078
With comorbidities		5	379	−0.48	−0.98, 0.03	81.7	0
Supplementation type	0.008						
Single‐type probiotics		5	455	−0.08	−0.27, 0.11	0	0.413
Multiple‐type probiotics		5	292	−0.73	−1.30, −0.15	79.7	0.001
Supplementation dosage	0.063						
< 10^9^ cfu		1	50	−0.94	−1.55, −0.32	NA	NA
10^9^ cfu ≤ Dose < 10^10^ cfu		3	238	−0.23	−0.53, 0.06	26.3	0.257
≥ 10^10^ cfu		6	459	−0.34	−0.77, 0.10	77.7	0
Duration of intervention	0.028						
≤ 8 weeks		2	80	−0.73	−1.21, 0.25	3.0	0.310
> 8 weeks		8	667	−0.26	−0.57, 0.04	71.8	0.001
Delivery format	0.439						
Dairy products		2	142	−0.43	−1.37, 0.52	84.7	0.010
Sachets or powder		2	156	−0.05	−0.37, 0.27	0	0.441
Capsules or tablets		6	449	−0.46	−0.90, −0.03	77.2	0.001
Quality of trials	0.244						
High quality		2	224	0.01	−0.27, 0.29	0	0.927
Moderate quality		4	248	−0.68	−1.73, 0.37	90.1	0
Low quality		4	275	−0.27	−0.51, −0.04	0	0.534

Abbreviations: BMI, body mass index; cfu, colony‐forming unit; CI, confidence interval; CRP, C‐reactive protein; NA, not applicable; SMD, standardized mean difference.

#### 3.3.5 Effects on Inflammatory Markers

There were 5 trials including 378 adults that indicated the pooled effect of probiotic administration on tumor necrosis factor‐alpha (TNF‐α), and 3 trials including 215 participants that reported Interleukin‐10 (IL‐10) concentrations. A nonsignificant increasing effect on TNF‐α, as well as IL‐10 was observed via probiotic intake (*n* = 5; SMD = 0.08; 95% CI: −0.60, 0.75; *p* = 0.826 and *n* = 3; SMD = 0.30; 95% CI: −0.53, 1.13; *p* = 0.479, respectively) (Figure 6). In spite of a highly severe statistical heterogeneity for TNF‐α and IL‐10 (*I* ^2^ = 89.5%; *p* = 0.000 and *I* ^2^ = 86.2%; *p* = 0.000, respectively), the subgroup analysis could not be executed, due to the limited number of trials (*n* = 5 and *n* = 3, respectively).

Four studies including 152 individuals demonstrated the pooled effect of probiotics on Interleukin‐6 (IL‐6) levels. A nonsignificant trivial lowering effect was observed via probiotic intake (*n* = 4; SMD = −0.09; 95% CI: −0.33, 0.14; *p* = 0.444) (Figure 6). Moreover, a mild statistical heterogeneity for IL‐6 (*I* ^2^ = 0%; *p* = 0.607) was revealed, and the subgroup analysis was not carried out, due to the limited number of trials (*n* = 4).

Zarrati et al. (2013) presented a significant decrease in IL‐4 and IL‐17 concentrations, accompanied by a significant rise in transforming growth factor‐beta (TGF‐β) levels after probiotic intake. Additionally, they showed a significant decrease in interferon‐gamma (IFN‐γ) levels, despite Schellekens et al. (2021) who reported no effects on this marker. Toshimitsu et al. (2021) revealed no significant changes in IL‐8 after supplementation, as well. We could not execute a meta‐analysis on IL‐4, IL‐17, IL‐8, and IFN‐γ, due to the limited number of studies (*n* < 3).

### 3.4 Findings From the Meta‐Regression Analysis

#### 3.4.1 Meta‐Regression Analysis Based on Supplementation Dosage

A meta‐regression analysis of SBP, DBP, glycemic control, lipid profile, CRP, and TNF‐α levels was conducted based on the supplementation dosage (Table 6).

**TABLE 6:** Meta‐regression of systolic and diastolic blood pressure, glycemic control, lipid profile, CRP, and TNF‐α based on probiotic dosage.

Parameter	Coefficient (CI)	*p*	Overall *I* ^2^ (%)	Residual *I* ^2^ (%)	Adjusted *R* ^2^ (%)
SBP	−2.80 × 10^−11^ (−4.57 × 10^−11^, −1.04 × 10^−11^)	0.004	85.2	85.7	45.87
DBP	−1.34 × 10^−11^ (−2.32 × 10^−11^, 0.35 × 10^−11^)	0.012	73.3	72.6	38.79
FPG	−1.47 × 10^−12^ (−4.21 × 10^−12^, 1.27 × 10^−12^)	0.273	0	0	0
HbA1c	−1.44 × 10^−12^ (−6.51 × 10^−12^, 3.63 × 10^−12^)	0.513	25.6	31.5	0
Fasting insulin	0.76 × 10^−12^ (−3.57 × 10^−12^, 5.09 × 10^−12^)	0.706	0	0	0
TC	−1.74 × 10^−12^ (−4.42 × 10^−12^, 0.92 × 10^−12^)	0.188	0.1	0	0
LDL	−1.17 × 10^−12^ (−3.86 × 10^−12^, 1.52 × 10^−12^)	0.373	0.2	1.1	0
HDL	−1.19 × 10^−12^ (−5.17 × 10^−12^, 2.79 × 10^−12^)	0.538	39.7	39.5	−5.75
TG	−0.35 × 10^−12^ (−5.09 × 10^−12^, 4.39 × 10^−12^)	0.878	23.9	27.8	0
CRP	−8.02 × 10^−12^ (−14.30 × 10^−12^, −1.78 × 10^−12^)	0.018	70.7	51.6	77.01
TNF‐α	−0.56 × 10^−10^ (−2.63 × 10^−10^, 1.50 × 10^−10^)	0.450	89.5	91.2	−8.81

Abbreviations: CI, confidence interval; CRP, C‐reactive protein; DBP, diastolic blood pressure; FPG, fasting plasma glucose; HbA1c, glycosylated hemoglobin; HDL, high‐density lipoprotein; LDL, low‐density lipoprotein; SBP, systolic blood pressure; TC, total cholesterol; TG, triglyceride; TNF‐α, t*umor necrosis factor*‐alpha.

Meta‐regression of the SBP and DBP demonstrated that the supplementation dosage plays a key role in the changes of effect size; since adjusted *R* ^2^ indicated a strong inverse relationship of the supplementation dosage with the levels of both SBP and DBP (adjusted *R* ^2^ = 45.87% and 38.79%, respectively) (Table 6 and Figure S81).

Furthermore, meta‐regression of the CRP revealed supplementation dosage as an important factor involved in the statistical heterogeneity (Table 6), as well as in the remarkable changes of effect size that indicated a strong inverse relationship of supplementation dosage with CRP concentrations (Adj *R* ^2^ = 77.01%) (Table 6 and Figure S82).

#### 3.4.2 Meta‐Regression Analysis Based on the Duration of the Intervention

A meta‐regression analysis of SBP and DBP, glycemic control, lipid profile, CRP, and TNF‐α levels was performed based on the duration of the intervention (Table 7). Meta‐regression results showed that the duration of the intervention is not a key factor involved in statistical heterogeneity, and as well, it does not play a significant role in effect size changes; since the results demonstrated no relationship between the supplementation duration and the aforementioned parameters (Table 7).

**TABLE 7:** Meta‐regression of systolic and diastolic blood pressure, glycemic control, lipid profile, CRP, and TNF‐α based on the duration of intervention.

Parameter	Coefficient (CI)	*p*	Overall *I* ^2^ (%)	Residual *I* ^2^ (%)	Adjusted *R* ^2^ (%)
SBP	7.15 × 10^−2^ (−3.67 × 10^−2^, 45.99 × 10^−2^)	0.697	85.2	86.1	−14.04
DBP	9.20 × 10^−2^ (−9.92 × 10^−2^, 28.34 × 10^−2^)	0.318	73.3	73.4	4.96
FPG	−4.18 × 10^−3^ (−54.7 × 10^−3^, 46.3 × 10^−3^)	0.863	0	0	0
HbA1c	4.52 × 10^−2^ (−5.16 × 10^−2^, 14.22 × 10^−2^)	0.297	25.6	22.3	0
Fasting insulin	2.46 × 10^−3^ (−66.19 × 10^−3^, 71.13 × 10^−3^)	0.938	0	0	0
TC	2.03 × 10^−2^ (−2.71 × 10^−2^, 6.78 × 10^−2^)	0.380	0.1	0.8	0
LDL	−3.78 × 10^−2^ (−0.95 × 10^−2^, 8.52 × 10^−2^)	0.111	0.2	0	0
HDL	−1.37 × 10^−2^ (−7.24 × 10^−2^, 4.50 × 10^−2^)	0.631	39.7	41.6	−6.32
TG	−2.47 × 10^−2^ (−8.23 × 10^−2^, 3.27 × 10^−2^)	0.378	23.9	24.8	0
CRP	5.12 × 10^−2^ (−3.29 × 10^−2^, 1.35 × 10^−2^)	0.198	70.7	68.1	23.09
TNF‐α	−24.22 × 10^−2^ (−90.75 × 10^−2^, 42.31 × 10^−2^)	0.330	89.5	89.9	8.36

Abbreviations: CI, confidence interval; CRP, C‐reactive protein; DBP, diastolic blood pressure; FPG, fasting plasma glucose; HbA1c, glycosylated hemoglobin; HDL, high‐density lipoprotein; LDL, low‐density lipoprotein; SBP, systolic blood pressure; TC, total cholesterol; TG, triglyceride; TNF‐α, t*umor necrosis factor*‐alpha.

### 3.5 Sensitivity Analysis

A one‐out‐remove sensitivity analysis was executed to explore the effect of each study on the overall effect size. The results of the one‐out‐remove method illustrated the robust findings of the present study.

### 3.6 Publication Bias

The visual inspection of funnel plot symmetry was investigated (Figures S83–S86), followed by Begg and Egger's tests in order to identify the possible publication bias. However, we did not evaluate the funnel plots of TNF‐α, IL‐6, IL‐10, and IFN‐γ; since there were not enough RCTs in the funnel plots related to these parameters (*n* < 10). According to Begg and Egger's tests, there was no evidence of publication bias regarding the FPG, fasting serum insulin, TNF‐α, IL‐6, IL‐10, and IFN‐γ parameters. Though there was evidence of slight to moderate asymmetry in funnel plots of SBP, DBP, and HbA1c, no publication bias regarding these parameters was revealed according to Begg and Egger's tests. As there was evidence of high publication bias in CRP levels based on the funnel plot, as well as Begg and Egger's tests, the trim and fill method was used to assess publication bias regarding this parameter accurately. However, the results of the trim and fill method indicated no publication bias related to CRP levels, as no studies were added by executing the trim and fill test.

### 3.7 Quality of Evidence

When comparing probiotic groups with controls, the quality of evidence for the outcomes of SBP and DBP was downgraded due to several factors. These included the risk of bias, severe inconsistency of effects among the included studies, and the presence of publication bias. Despite the presence of a dose–response relationship in the primary studies, which would typically upgrade the quality of evidence for these outcomes, the overall quality of evidence was ultimately assessed as very low for SBP and DBP (Table 8).

**TABLE 8:** Quality of evidence of primary outcomes according to the GRADE approach.

Outcome	No. of studies	Downgrading factors				Upgrading factors			Quality per grade
		Risk of bias	Inconsistency	Imprecision	Publication bias	Magnitude of effect	Dose–response relationship	Plausible confounders	
SBP	15	Serious (−1)	Very serious (−2)	Not serious	Serious (−1)	Trivial	Yes (+1)	No	Very low
DBP	15	Serious (−1)	Serious (−1)	Not serious	Serious (−1)	Low‐sized	Yes (+1)	No	Low
FPG	18	Very serious (−2)	Not serious	Not serious	Not serious	Trivial	No	No	Low
HbA1c	8	Very serious (−2)	Not serious	Not serious	Serious (−1)	Trivial	No	No	Very low
Fasting insulin	13	Very serious (−2)	Not serious	Not serious	Not serious	Low‐sized	No	No	Low
TC	21	Very serious (−2)	Not serious	Not serious	Not serious	Trivial	No	No	Low
LDL	21	Very serious (−2)	Not serious	Not serious	Not serious	Trivial	No	No	Low
HDL	21	Very serious (−2)	Not serious	Not serious	Not serious	Trivial	No	No	Low
TG	20	Very serious (−2)	Not serious	Not serious	Not serious	Trivial	No	No	Low
CRP	10	Serious (−1)	Serious (−1)	Not serious	Serious (−1)	Low‐sized	Yes (+1)	No	Low
TNF‐α	5	Very serious (−2)	Very serious (−2)	Not serious	Not serious	Trivial	No	Yes (+1)	Low
IL‐6	4	Not serious	Not serious	Serious (−1)	Not serious	Trivial	No	Yes (+1)	High
IL‐10	3	Very serious (−2)	Very serious (−2)	Very serious (−2)	Not serious	Low‐sized	No	Yes (+1)	Very low
IFN‐γ	2	Serious (−1)	Serious (−1)	Very serious (−2)	Not serious	Low‐sized	No	Yes (+1)	Very low

*Note:* High certainty: We are very confident that the true effect lies close to that of the estimate of the effect. Moderate certainty: Further research is likely to affect our confidence in the effect estimate and may change the estimate. Low certainty: We are not confident in the estimate of effect and the true value might be different from it. Very low certainty: We do not have any confidence in the estimate of effect and it is likely that the true value is considerably different from it.

Abbreviations: CRP, C‐reactive protein; DBP, diastolic blood pressure; FPG, fasting plasma glucose; HbA1c, glycosylated hemoglobin; HDL, high‐density lipoprotein; IFN‐γ, interferon‐gamma; IL, interleukin; LDL, low‐density lipoprotein; SBP, systolic blood pressure; TC, total cholesterol; TG, triglyceride; TNF‐α, t*umor necrosis factor*‐alpha.

Moreover, there was a very low overall quality of evidence for the outcome of HbA1c because of evidence for a severe risk of bias, as well as publication bias. A low overall quality of evidence was also observed for FPG and serum insulin, due to the severe risk of bias regarding these outcomes (Table 8).

When comparing probiotic groups with controls, the quality of evidence for lipid profile outcomes, including TC, LDL‐C, HDL‐C, and TG, was downgraded. This was primarily due to the severe risk of bias present in the included trials. Consequently, a low overall quality of evidence regarding lipid profile markers was identified (Table 8).

The quality of evidence for the outcomes of CRP and inflammatory markers was also assessed. Despite an increase in the quality of evidence for CRP due to the presence of a dose–response relationship in the primary studies, a low overall quality of evidence regarding this parameter was observed. This was primarily due to the presence of risk of bias, as well as publication bias in the included trials (Table 8).

Additionally, while there was a high overall quality of evidence for interleukin‐6 (IL‐6), a low quality of evidence for the outcomes of TNF‐α was observed. This downgrade in quality was attributed to the severe risk of bias and inconsistency of effects observed in the primary studies (Table 8). Finally, our findings indicated a very low overall quality of evidence for IL‐10 and IFN‐γ outcomes. This was due to the risk of bias, severe inconsistency of effects, and imprecision around the effect estimate (Table 8).

## 4 Discussion

To our knowledge, this systematic review and meta‐analysis is the first to provide a combination of evidence that presents the effect of probiotics, with a precise exclusion of prebiotics, on CVD risk factors in overweight and obesity. Clinical and epidemiological studies have revealed a strong link between obesity and a vast spectrum of CVD risk factors including elevated BP, impaired lipid profile, glucose intolerance, T2DM, and raised inflammatory markers (Wilkins et al. 2017; World Health Organization 2007; Wilson et al. 2002). Similar to the previous studies (Lim et al. 2020; Schellekens et al. 2021; Zarrati et al. 2013), our findings suggest that probiotics could have ameliorating effects on BP, glycemic control, blood lipids, inflammatory parameters, and CRP in overweight and obese individuals.

Hypertension is a serious risk factor for CVD (Nguyen et al. 2020). Although our study showed a trivial to low‐sized reducing effect of probiotic administration on SBP and DBP, it should be considered that even a small decrease in BP may lead to a remarkable reduction in the incidence of stroke and CVD (Daliri et al. 2017). Our findings revealed that the best results were detected in people with comorbidities, those who used probiotics for more than 8 weeks, individuals supplemented by a dosage of 10^10^ cfu or more, adults who consumed multiple types of probiotics, and those who used capsules and tablets as means of administration instead of probiotic‐fermented products. The results were in agreement with several previous meta‐analyses, which reported a slight to moderate reduction in SBP and DBP after probiotic supplementation (Hendijani and Akbari 2018; da Silva Pontes et al. 2021; Lewis‐Mikhael et al. 2020; Ejtahed et al. 2020). Although Hendijani and Akbari (2018) demonstrated larger lowering effects in their meta‐analysis than this review, it should be noted that their meta‐analysis was conducted on diabetic patients while the participants of the present review were overweight or obese. Besides, similar to Hendijani and Akbari (2018), we observed a large‐sized decrease in people with comorbidities, such as elevated FPG concentrations. On the other hand, they reported that probiotic intake led to less significant effects on BP in more obese participants. It is, therefore, possible that one of the reasons resulting in smaller changes of SBP and DBP in our study might be due to differences in anthropometric characteristics of adults who had higher levels of BMI, compared with the meta‐analysis of Hendijani and Akbari (2018). Another possible reason might be due to the impaired intestinal microbiome in obese people that may weaken the effect of supplementation, because probiotics are supposed to modulate the intestinal flora prior to BP normalization.

Similar to the current review, Hendijani and Akbari and Ejtahed et al. revealed larger lowering effects on BP through multi‐strain probiotic consumption (Hendijani and Akbari 2018; Ejtahed et al. 2020). However, in contrast with our results, Mayta‐Tovalino et al. (2023) showed no changes in BP by consuming probiotics. These contradictory findings obtained by different meta‐analyses could be attributed to differences in the characteristics of the study population, dosage and duration of the intervention, and probiotic strains. Furthermore, the results regarding the meta‐regression demonstrated a strong inverse relationship between the probiotic dosage with SBP and DBP. Hence, it seems that treatment at a higher dosage (≥ 10^10^ cfu) of probiotics could lead to more beneficial impacts on the SBP and DBP of overweight and obese individuals than a low or moderate dosage (< 10^10^ cfu). However, it seems necessary to re‐evaluate these results in future meta‐analyses in order to obtain more accurate findings.

Since a large degree of statistical heterogeneity was observed for the variations in BP, we executed subgroup analysis to identify the possible sources of heterogeneity. The statistical heterogeneity was significantly lower in healthy adults, those using probiotic dosages < 10^9^ cfu, and participants who received probiotic‐fermented dairy products. Therefore, we considered that the supplementation dosage and means of administration, as well as the health condition, might be sources of heterogeneity in the associated RCTs.

Although the specific mechanism of action involved in the antihypertensive impacts of probiotics has yet to be completely clarified, the general effect on improving BP is probably via two major mechanisms: (1) Vasodilation effect: Probiotics are known to produce short‐chain fatty acids (SCFAs) from fermenting nondigestible carbohydrates. These metabolites can be absorbed into the bloodstream and then attach to special receptors called G‐protein coupled receptors (GPCRs). It has been shown that these receptors have hypotensive effects (Méndez‐Albiñana et al. 2022). Besides, probiotic‐derived metabolites could help in the biosynthesis of angiotensin‐converting enzyme (ACE) inhibitors, nitric oxide, and prostaglandin *I* ^2^, which are prominent factors in inducing vasodilation and BP reduction (Fitzgerald and Murray 2006; Ahrén et al. 2015; Aoyagi et al. 2017). (2) Protection of endothelial integrity: Probiotics have profound effects on preventing endothelial damage through decreasing lipid peroxidation and oxidative stress (Noce et al. 2019; Sircana et al. 2019). It is also stated that they reduce vascular inflammation via modulation of the TH17 cells, which produce IL‐17 (Wilck et al. 2017). Metabolic diseases can cause dysbiosis and increase the Firmicutes/Bacteroidetes proportion in the gut. The lipopolysaccharides from bacteria can translocate into the blood and cause low‐grade inflammation. Probiotics may lower the levels of this endotoxemia and prevent inflammation (Méndez‐Albiñana et al. 2022). The improving effects of probiotics on insulin sensitivity and inhibition of endothelium glycation are another way to prevent endothelial dysfunction (Lye et al. 2009; Toral et al. 2014).

Results from our meta‐analysis revealed that probiotic consumption could be effective in the promotion of glucose metabolism. A slight, yet significant effect was observed on FPG and fasting insulin concentrations, but not on HbA1c levels. In line with the present review, Wang et al. and da Silva Pontes et al. reported significant impacts of probiotics on FPG and fasting insulin accompanied by insignificant changes in glycosylated hemoglobin levels in overweight and obese participants (da Silva Pontes et al. 2021; Wang et al. 2019). Although subgroup analysis illustrated a small‐sized decreasing effect of probiotics on HbA1c level in trials with a duration of 8 weeks or less, the results are not reliable owing to the limited number of trials (*n* = 3). Furthermore, according to the subgroup analysis results, probiotics may affect FPG and serum insulin effectively in healthy people, those using supplementation dosages of 10^10^ cfu or more, and those consuming capsules or tablets as their delivery format.

It is suggested that the mechanism of action through which probiotics prevent insulin resistance is via the inhibition of the β‐cell destruction in the islets of Langerhans, promoting the transcription of glucose transporter 4 (GLUT4) and extracellular signal‐regulated kinase 2 (ERK2) levels, as well as alleviating inflammation (Valenlia et al. 2018). Additionally, SCFAs produced by probiotics may decrease insulin resistance by increasing glucagon‐like peptide‐1 (GLP‐1) and peptide YY (PYY) (Zhai et al. 2021). Evidence suggests that insulin receptor substrate‐phosphatidylinositol 3‐OH kinase (IRSPI3K) may conduct a hypothalamic signal transduction, which is a key determinant of peripheral tissues' insulin sensitivity. Probiotic byproducts stimulate glucose uptake and metabolism in the brain and peripheral tissues; consequently, improving glycemic indices (Morton et al. 2005).

Abnormal lipid profiles, particularly increased concentrations of TC and LDL‐C, have been suggested as the major risk factors for CVD (Cho and Kim 2015). Our results indicated slight but statistically significant beneficial effects of probiotic intake on serum lipids. In line with our findings, Wang et al. and da Silva Pontes et al. mentioned significant trivial impacts of probiotics on blood lipids in overweight and obese adults (Wang et al. 2021; da Silva Pontes et al. 2021). However, there are other previous studies, including a systematic review and meta‐analysis by Yan et al. that reported larger effects on lipid profile markers, especially on TC and LDL‐C concentrations (Wang et al. 2019; Yan et al. 2019). The observed discrepancy between the findings of the present review and previous studies may be due to the differences in the characteristics of participants, intervention dosage, probiotic strains, and type of probiotic delivery. Furthermore, it should be considered that Yan et al. investigated the effects of probiotics in a wide age range and reported larger reductions of TC and LDL‐C in children and adolescents than in adults. In other words, it seems that higher baseline cholesterol concentrations in adults make it difficult to address high cholesterol, in comparison to children.

According to our subgroup analysis, the beneficial effects of probiotic intake on lipid profile were mostly in individuals with comorbidities, those who were supplemented for 8 weeks or less, and those who used probiotic‐fermented dairy products as their delivery format. Although Hendijani and Akbari indicated larger improvements in lipid profile via the consumption of higher probiotic dosage (Hendijani and Akbari 2018), no differences were observed in the results regarding subgroup analysis based on probiotic dosage in the present study. Moreover, according to the findings obtained by Yan et al. and Hendijani and Akbari, probiotics may lead to stronger improvements in blood lipids via multi‐strain probiotics, rather than single‐strain types (Hendijani and Akbari 2018; Yan et al. 2019); while in the present meta‐analysis, no differences were revealed between participants who consumed single‐strain probiotics and those who used multi‐strain types. Nevertheless, it is probable that the simultaneous use of various strains may result in cooperative and synergistic interactions between different probiotic strains. In addition, similar to Hendijani and Akbari (2018), our subgroup analysis demonstrated larger incremental effects of probiotics on HDL‐C concentrations in obese participants, rather than overweight individuals, which might occur owing to restoring balance and harmony to the intestinal microbiome in obese and dyslipidemic participants via consumption of probiotics. On the other hand, in contrast to Hendijani and Akbari, our results indicated smaller lowering impacts of probiotics on TG levels in obese people, which may be due to the smaller sample size and limited trials, as wider CI were observed.

Due to the moderate‐to‐severe magnitude of the heterogeneity in the variations of blood lipids in the current study, a subgroup analysis was executed. The statistical heterogeneity was significantly lower in people older than 42 years, participants who consumed single‐type probiotics, adults who used probiotic capsules, tablets, or sachets instead of probiotic‐fermented products, and trials with a duration of more than 8 weeks. Therefore, we considered that the age of participants, types of administered probiotics, delivery format, and treatment duration might be sources of heterogeneity in the primary studies.

Indeed, the mechanisms underlying the beneficial effects of probiotics on lipid metabolism predominantly involve their role in reducing the enterohepatic circulation of bile salts. For instance, certain probiotics, such as Bifidobacterium spp., exhibit resistance to bile acids through the production of bile salt hydrolases (BSH). These enzymes deconjugate glycine or taurine in the steroid core of bile salts, leading to their degradation. Consequently, the liver increases cholesterol mobilization to regenerate bile salts (Yan et al. 2019). Overall, BSH activity has been linked to systemic metabolic benefits, including a reduction in plasma cholesterol concentration, hepatic triglyceride levels, and weight gain (Costabile et al. 2017; Suez et al. 2019). Additionally, probiotics may induce satiety by producing SCFAs and possess the ability to bind lipid molecules, thereby inhibiting their intestinal absorption (Gilliland et al. 1985).

In this review, probiotic administration indicated an insignificant incremental effect on TNF‐α and IL‐10 levels in healthy people. However, it should be considered that our findings were from a meta‐analysis of five and three primary trials, respectively. In addition, after investigating the results of the included studies, we found that the increasing effects of probiotics on TNF‐α and IL‐10 levels were owing to a trial performed by Zarrati et al. (2013). They reported larger reductions of these parameters in control subjects, in comparison to the treatment group, and therefore, it seems necessary to re‐evaluate the findings related to inflammatory markers in future meta‐analyses with enough numbers of included RCTs. Furthermore, a slight lowering impact of probiotic intake on IL‐6 was observed, which could not be reliable due to the insufficient number of included trials (*n* = 4).

Our findings indicated a remarkably low‐sized reducing effect of probiotics on CRP levels. This result was similar to that of the previous study by Kazemi et al. which demonstrated lower levels of serum CRP in healthy adults and patients with metabolic disorders supplemented with probiotics (Kazemi et al. 2020). Also, in agreement with the current meta‐analysis, da Silva Pontes et al. (2021) reported a small‐sized reducing effect of probiotic administration on CRP concentrations in overweight and obese adults. Finally, we carried out a meta‐regression test, which indicated a significant inverse relationship between the supplementation dosage and CRP concentrations. Thus, it is probable that a higher probiotic dosage (> 10^10^ cfu) could be more effective in reducing the CRP level of overweight and obese people.

Probiotic administration represents a promising approach to mitigate low‐grade inflammation, characterized by a reduction in pro‐inflammatory markers such as TNF‐α and IL‐6, alongside an increase in anti‐inflammatory markers like adipose‐derived adiponectin. This modulation may also contribute to lower concentrations of CRP (Zarrati et al. 2014; Torres et al. 2019; Bernini et al. 2016). Generally, the anti‐inflammatory effects of probiotics are thought to occur through various mechanisms, including (a) regulation of the maturation of dendritic cells, as well as the expression of toll‐like receptors (TLRs) in the intestine. (b) Increasing the production of anti‐inflammatory cytokines from regulatory T‐cells (Tregs). (c) Induction of the differentiation of T‐helper cells into Th2 cells (Torres et al. 2019).

In the current meta‐analysis, to ensure consistency across various outcome assessment methods employed in different trials, the SMD was calculated using Glass's delta method to estimate the effect size. SMD provides information on statistical units and does not give any information on clinical units. Therefore, it is difficult to clinically interpret the intervention effect when expressed as SMD, since it is reported in SD units rather than in units of each measurement scale. Based on SMD, small changes were observed in the outcomes studied. While these changes are relatively small, they could have significant implications for individuals with prediabetes or metabolic syndrome, potentially delaying or preventing the onset of type 2 diabetes. These small changes in cardiometabolic factors can accumulate and collectively have beneficial effects on health. Overall, the results align with the increasing emphasis on lifestyle and dietary interventions in diabetes management guidelines.

The results of the GRADE approach indicated that the evidence generated by the meta‐analysis for SBP and DBP was of very low and low quality, respectively. The main reasons for downgrading the quality of evidence related to SBP and DBP were the risk of bias, inconsistency of effects, and publication bias in the primary studies. A low quality of evidence was also revealed for the outcomes of glycemic indices and lipid profiles. The risk of bias in the primary trials was the primary factor downgrading the quality of evidence regarding the glycemic indices, as well as lipid profile parameters. Finally, a low or very low quality of evidence was observed for the outcomes of CRP and inflammatory parameters, except for interleukin‐6 (IL‐6). The reasons for downgrading the quality of evidence for CRP and inflammatory markers were mainly due to the risk of bias and inconsistency of effects in the primary trials. Additionally, despite the high quality of evidence for the IL‐6 outcome, the result is not entirely reliable due to the limited number of studies (*n* = 4).

## 5 Strengths and Limitations

The main strength of the current systematic review and meta‐analysis was that we excluded the trials in which the pure effects of probiotics from combined treatments such as synbiotics could not be distinguished. In addition, a comprehensive subgroup analysis was conducted and the results were stratified by some relevant variables, including the baseline characteristics of the studied populations, as well as the supplementation dosage and duration. A meta‐regression test was also performed to determine the optimum probiotic supplementation dosage and duration in overweight and obesity. Finally, it should be noted that all stages of the systematic review were conducted by four independent reviewers and no language restrictions were set in order to achieve more accurate results.

However, there were also some limitations in this study. First of all, our analysis included studies up to March 31, 2023, following our pre‐registered protocol. Although more recent studies may have been published since our initial search, we did not update it during the revision process. However, given our comprehensive review, an update is unlikely to change the conclusions. Furthermore, due to the insufficient number of primary trials, we could not execute subgroup analysis according to the strains of probiotics. In addition, no reliable microbiological tests were performed by the included trials to confirm the viability of microorganisms. Hence, we could not investigate the relationship between gut microbiota composition and changes in CVD risk factors in overweight or obese individuals.

## 6 Implications

Our findings suggest that probiotics can serve as a valuable adjunct to conventional therapies for managing metabolic and inflammatory parameters in overweight and obese individuals. Clinicians could consider recommending probiotic supplementation as part of a comprehensive treatment plan for overweight and obese patients, particularly those at risk of metabolic syndrome. In this regard, probiotics should be incorporated alongside lifestyle interventions, including diet and exercise. In addition, strains with proven efficacy (e.g., *Lactobacillus* and *Bifidobacterium* species) should be prioritized. Further research is needed to determine the most effective strains, dosages, and treatment durations, as well as to explore the long‐term impacts of probiotics on clinical outcomes in overweight and obese populations.

## 7 Conclusion

In summary, this systematic review and meta‐analysis indicate that probiotic supplementation in overweight and obese individuals can lead to reductions in SBP and DBP, improvements in glucose and lipid metabolism, and decreases in CRP concentrations. Subgroup analysis suggests that probiotic intake may effectively impact these parameters in individuals with comorbidities, those receiving higher intervention dosages, and those using probiotic capsules or tablets as opposed to other forms of administration. Additionally, a reverse dose–response relationship was observed between supplementation dosage and BP, as well as CRP levels. Furthermore, given that effect sizes in this meta‐analysis were expressed using SMD, the clinical significance of the findings is difficult to interpret since it was reported in SD units, rather than in units of each measurement scale. In other words, SMD provides information on statistical units and cannot give any information on clinical units. Overall, further clinical trials and meta‐analyses are necessary to definitively recommend probiotics as a non‐pharmacologic alternative for preventing CVD risk factors in overweight and obese individuals.

## Author Contributions

**Safa Sefidgari‐Abrasi:** data curation (equal), formal analysis (equal), writing – review and editing (equal). **Marziyeh Rahimiyan‐Heravan:** conceptualization (equal), investigation (equal), validation (equal), methodology (equal). **Vahid Amiran:** conceptualization (equal), investigation (equal), methodology (equal). **Elham Navval‐Esfahlan:** methodology (equal), validation (equal). **Maryam Saghafi‐Asl:** supervision (equal).

## Conflicts of Interest

The authors declare no conflicts of interest.

## Supporting information


**TABLE S1.** Electronic search strategy.
**TABLE S2.** Primary studies' quality (risk of bias).
**FIGURE S1–S8.** Forest plot displaying subgroup analysis of the outcome of systolic blood pressure based on participants' and treatment characteristics.
**FIGURE S9–S16.** Forest plot displaying subgroup analysis of the outcome of diastolic blood pressure based on participants' and treatment characteristics.
**FIGURE S17–S24.** Forest plot displaying subgroup analysis of the outcome of fasting plasma glucose based on participants' and treatment characteristics.
**FIGURE S25–S32.** Forest plot displaying subgroup analysis of the outcome of hemoglobin A1C based on participants' and treatment characteristics.
**FIGURE S33–S40.** Forest plot displaying subgroup analysis of the outcome of fasting serum insulin based on participants' and treatment characteristics.
**FIGURE S41–S48.** Forest plot displaying subgroup analysis of the outcome of total cholesterol based on participants' and treatment characteristics.
**FIGURE S49–S56.** Forest plot displaying subgroup analysis of the outcome of low‐density lipoprotein cholesterol based on participants' and treatment characteristics.
**FIGURE S57–S64.** Forest plot displaying subgroup analysis of the outcome of high‐density lipoprotein cholesterol based on participants' and treatment characteristics.
**FIGURE S65–S72.** Forest plot displaying subgroup analysis of the outcome of serum triglyceride based on participants' and treatment characteristics.
**FIGURE S73–S80.** Forest plot displaying subgroup analysis of the outcome of C‐reactive protein based on participants' and treatment characteristics.
**FIGURE S81.** Meta‐regression analysis of the (a) systolic and (b) diastolic blood pressure based on supplementation dosage.
**FIGURE S82.** Meta‐regression analysis of the C‐reactive protein based on supplementation dosage.
**FIGURE S83.** Funnel plot displaying the publication bias of the included trials reporting the effects of probiotics on (a) systolic and (b) diastolic blood pressure.
**FIGURE S84.** Funnel plot displaying the publication bias of the included trials reporting the effects of probiotics on (a) fasting plasma glucose, (b) hemoglobin A1C, and (c) fasting insulin.
**FIGURE S85.** Funnel plot displaying the publication bias of the included trials reporting the effects of probiotics on (a) total cholesterol, (b) low‐density lipoprotein, (c) high‐density lipoprotein, and (d) triglyceride.
**FIGURE S86.** Funnel plot displaying the publication bias of the included trials reporting the effects of probiotics on C‐reactive protein.

## Data Availability

The datasets used and/or analyzed during the present systematic review and meta‐analysis are available from the corresponding author upon request.
