# Correction to “The Effects of Probiotics Consumption on Blood Pressure, Lipid Profile, Glycemic Indices, and Inflammatory Parameters in Overweight and Obese Adults: A Systematic Review and Meta‐Analysis of Randomized Controlled Trials”

**DOI:** 10.1002/fsn3.70874

**Published:** 2025-08-26

Sefidgari‐Abrasi, S., M. Rahimiyan‐Heravan, V. Amiran, E. Navval‐Esfahlan, and M. Saghafi‐Asl. 2025. “The Effects of Probiotics Consumption on Blood Pressure, Lipid Profile, Glycemic Indices, and Inflammatory Parameters in Overweight and Obese Adults: A Systematic Review and Meta‐Analysis of Randomized Controlled Trials.” *Food Science & Nutrition* 13, no. 8: e70434. https://doi.org/10.1002/fsn3.70434.

In the originally published version, the following corrections are noted:

In the text, *p* = 0.000 was erroneously written instead of *p* < 0.001. The corrected text appears here:

- Section 3.3.1.: Heterogeneity test results also showed a highly severe heterogeneity (*I* ^2^ = 85.2%; *p* < 0.001).
- Section 3.3.1: Besides, heterogeneity test results showed severe statistical heterogeneity (*I* ^2^ = 73.3%; *p* < 0.001). Section 3.3.4: Heterogeneity test results presented severe statistical heterogeneity (*I* ^2^ = 70.7%; *p* < 0.001)
- Section 3.3.5.: In spite of a highly severe statistical heterogeneity for TNF‐α and IL‐10 (*I* ^2^ = 89.5%; *p* < 0.001 and *I* ^2^ = 86.2%; *p* < 0.001, respectively), the subgroup analysis could not be executed due to the limited number of trials (*n* = 5 and *n* = 3, respectively).

In section 3.3.1., “We conducted subgroup analysis for SBP and DBP based on age, gender, BMI, health condition, delivery format, as well as supplementation type, dosage, and duration (Table 2 and Figure S1)” should be corrected to “We conducted subgroup analysis for SBP and DBP based on age, gender, BMI, health condition, delivery format, as well as supplementation type, dosage, and duration (Table 2 and Figures S1–S8).”

In section 3.3.3., “Twenty‐one trials including 1602 participants presented the pooled effect of probiotic administration on TC, LDL‐C, and HDL‐C” should be corrected to “Twenty‐one trials including 1602 participants presented the data of probiotic administration on TC, LDL‐C, and HDL‐C.”

The online version of the article has been corrected accordingly.

We apologize for these errors.

