# Supplementary material for: The Effects of Probiotics Consumption on Blood Pressure, Lipid Profile, Glycemic Indices, and Inflammatory Parameters in Overweight and Obese Adults: A Systematic Review and Meta‐Analysis of Randomized Controlled Trials
Source: Food Sci Nutr. 2025 Jul 27;13(8):e70434. doi: 10.1002/fsn3.70434 (PMC12301575; doi:10.1002/fsn3.70434)

| Supp Table 1. Electronic Search Strategy | | | | |
| --- | --- | --- | --- | --- |
| **Database**  **(date of search)** | **Strategy** | **Official Literature** | **Gray Literature** | **Total** |
| Pubmed/Medline | (“body weight”[tiab] OR (weight*[tiab] AND body[tiab]) OR overweight[tiab] OR obesity[tiab] OR obese[tiab]) AND (lactobacillus[tiab] OR bifidobacterium[tiab] OR bifidobacteria[tiab] OR "lactic acid bacteria"[tiab] OR “lactobacillus acidophilus”[tiab] OR acidophilus[tiab] OR “lactobacillus casei”[tiab] OR casei[tiab] OR “Lactobacillus plantarum”[tiab] OR Plantarum[tiab] OR Carecne[tiab] OR Aldicom[tiab] OR “Saccharomyces boulardii”[tiab] OR Bioflor[tiab] OR Bioflora[tiab] OR Codex[tiab] OR Econorm[tiab] OR Enflor[tiab] OR Enterol[tiab] OR Florastor[tiab] OR Florestor[tiab] OR Perenterol[tiab] OR Perenteryl[tiab] OR Precosa[tiab] OR Reflor[tiab] OR “ultra levure”[tiab] OR ultralevure[tiab] OR probiotic[tiab] OR probiotics[tiab]) AND 1991/01/01:2023/03/31[dp] | 5003 |  | 5003 |
| Scopus | (TITLE-ABS("body weight") OR (TITLE-ABS(weight*) AND TITLE-ABS(body)) OR TITLE-ABS(overweight) OR TITLE-ABS(obesity) OR TITLE-ABS(obese)) AND (TITLE-ABS(lactobacillus) OR TITLE-ABS(Bifidobacterium) OR TITLE-ABS(bifidobacteria) OR TITLE-ABS("lactic acid bacteria") OR TITLE-ABS("lactobacillus acidophilus") OR TITLE-ABS(acidophilus) OR TITLE-ABS("lactobacillus casei") OR TITLE-ABS(casei) OR TITLE-ABS("Lactobacillus plantarum") OR TITLE-ABS(Plantarum) OR TITLE-ABS(Carecne) OR TITLE-ABS(Aldicom) OR TITLE-ABS("Saccharomyces boulardii") OR TITLE-ABS(Bioflor) OR TITLE-ABS(Bioflora) OR TITLE-ABS(Codex) OR TITLE-ABS(Econorm) OR TITLE-ABS(Enflor) OR TITLE-ABS(Enterol) OR TITLE-ABS(Florastor) OR TITLE-ABS(Florestor) OR TITLE-ABS(Perenterol) OR TITLE-ABS(Perenteryl) OR TITLE-ABS(Precosa) OR TITLE-ABS(Reflor) OR TITLE-ABS("ultra levure") OR TITLE-ABS(ultralevure) OR TITLE-ABS(probiotic) OR TITLE-ABS(probiotics)) AND PUBYEAR > 1990 AND PUBYEAR < 2024 AND NOT PUBDATETXT("April 2023" OR "May 2023" OR "June 2023" OR "July 2023" OR "August 2023" OR "September 2023" OR "October 2023" OR "November 2023" OR "December 2023") | 7085 | 122 | 7207 |
| Web of Science | (TS=("body weight") OR (TS=(weight*) AND TS=(body)) OR TS=(overweight) OR TS=(obesity) OR TS=(obese)) AND (TS=(lactobacillus) OR TS=(Bifidobacterium) OR TS=(bifidobacteria) OR TS=("lactic acid bacteria") OR TS=(“lactobacillus acidophilus”) OR TS=(acidophilus) OR TS=(“lactobacillus casei”) OR TS=(casei) OR TS=(“Lactobacillus plantarum”) OR TS=(Plantarum) OR TS=(Carecne) OR TS=(Aldicom) OR TS=(“Saccharomyces boulardii”) OR TS=(Bioflor) OR TS=(Bioflora) OR TS=(Codex) OR TS=(Econorm) OR TS=(Enflor) OR TS=(Enterol) OR TS=(Florastor) OR TS=(Florestor) OR TS=(Perenterol) OR TS=(Perenteryl) OR TS=(Precosa) OR TS=(Reflor) OR TS=(“ultra levure”) OR TS=(ultralevure) OR TS=(probiotic) OR TS=(probiotics)) AND PY=(1991-2023) | 11922 | 367 | 12289 |
| EMBASE | (‘body weight’:ti,ab OR (weight*:ti,ab AND body:ti,ab) OR overweight:ti,ab OR obesity:ti,ab OR obese:ti,ab) AND (lactobacillus:ti,ab OR Bifidobacterium:ti,ab OR bifidobacteria:ti,ab OR ‘lactic acid bacteria’:ti,ab OR ‘lactobacillus acidophilus’:ti,ab OR acidophilus:ti,ab OR ‘lactobacillus casei’:ti,ab OR casei:ti,ab OR ‘Lactobacillus plantarum’:ti,ab OR Plantarum:ti,ab OR Carecne:ti,ab OR Aldicom:ti,ab OR ‘Saccharomyces boulardii’:ti,ab OR Bioflor:ti,ab OR Bioflora:ti,ab OR Codex:ti,ab OR Econorm:ti,ab OR Enflor:ti,ab OR Enterol:ti,ab OR Florastor:ti,ab OR Florestor:ti,ab OR Perenterol:ti,ab OR Perenteryl:ti,ab OR Precosa:ti,ab OR Reflor:ti,ab OR ‘ultra levure’:ti,ab OR ultralevure:ti,ab OR probiotic:ti,ab OR probiotics:ti,ab) AND [1991-2023]/py | 4839 | 1123 | 5962 |
| CENTRAL | (‘body weight’:ti,ab OR (weight*:ti,ab AND body:ti,ab) OR overweight:ti,ab OR obesity:ti,ab OR obese:ti,ab) AND (lactobacillus:ti,ab OR Bifidobacterium:ti,ab OR bifidobacteria:ti,ab OR ‘lactic acid bacteria’:ti,ab OR ‘lactobacillus acidophilus’:ti,ab OR acidophilus:ti,ab OR ‘lactobacillus casei’:ti,ab OR casei:ti,ab OR ‘Lactobacillus plantarum’:ti,ab OR Plantarum:ti,ab OR Carecne:ti,ab OR Aldicom:ti,ab OR ‘Saccharomyces boulardii’:ti,ab OR Bioflor:ti,ab OR Bioflora:ti,ab OR Codex:ti,ab OR Econorm:ti,ab OR Enflor:ti,ab OR Enterol:ti,ab OR Florastor:ti,ab OR Florestor:ti,ab OR Perenterol:ti,ab OR Perenteryl:ti,ab OR Precosa:ti,ab OR Reflor:ti,ab OR ‘ultra levure’:ti,ab OR ultralevure:ti,ab OR probiotic:ti,ab OR probiotics:ti,ab) | 1152 |  | 1152 |
| ProQuest | (AB,TI("body weight") OR (AB,TI(weight*) AND AB,TI(body)) OR AB,TI(overweight) OR AB,TI(obesity) OR AB,TI(obese)) AND (AB,TI(lactobacillus) OR AB,TI(Bifidobacterium) OR AB,TI(bifidobacteria) OR AB,TI("lactic acid bacteria") OR AB,TI(“lactobacillus acidophilus”) OR AB,TI(acidophilus) OR AB,TI(“lactobacillus casei”) OR AB,TI(casei) OR AB,TI(“Lactobacillus plantarum”) OR AB,TI(Plantarum) OR AB,TI(Carecne) OR AB,TI(Aldicom) OR AB,TI(“Saccharomyces boulardii”) OR AB,TI(Bioflor) OR AB,TI(Bioflora) OR AB,TI(Codex) OR AB,TI(Econorm) OR AB,TI(Enflor) OR AB,TI(Enterol) OR AB,TI(Florastor) OR AB,TI(Florestor) OR AB,TI(Perenterol) OR AB,TI(Perenteryl) OR AB,TI(Precosa) OR AB,TI(Reflor) OR AB,TI(“ultra levure”) OR AB,TI(ultralevure) OR AB,TI(probiotic) OR AB,TI(probiotics)) AND YR(19910101-20230331) |  | 261 | 261 |
| ClinicalTrials.gov |  | 5 | 0 | 5 |
| ISRCTN Registry |  | 0 | 0 | 0 |
| ICTRP |  | 4 | 2 | 6 |
| **TOTAL** |  | **30,010** | **1875** | **31885** |

| Supp Table 2. Primary studies’ quality (Risk of bias) | | | | | | | | | |
| --- | --- | --- | --- | --- | --- | --- | --- | --- | --- |
| First author, Year | Sequence generation* | | Allocation sequence concealment | Single blinding* | Double blinding | Incomplete outcome data* | Selective outcome reporting | Similarity at the baseline | Quality status |
| Agerholm-Larsen, 2000 (50) | | Unclear | Unclear | Unclear | Low risk | High risk | Low risk | High risk | Low |
| Cho 2022 (33) | | Unclear | Unclear | Unclear | Unclear | High risk | Low risk | Low risk | Low |
| Choi 2022 (30) | | Low risk | Low risk | Low risk | Low risk | Low risk | Low risk | Low risk | High |
| Crovesy de Oliveira 2020 (36) | | Low risk | Low risk | Low risk | Low risk | High risk | Low risk | High risk | Moderate |
| Déchelotte 2021 (60) | | Unclear | Unclear | Unclear | Unclear | Low risk | Low risk | Low risk | Low |
| Gomes, 2017 (51) | | Low risk | Low risk | Unclear | Low risk | Unclear | High risk | High risk | Low |
| Hajipoor, 2021 (31) and Hajipoor 2020 (61) | | Low risk | Low risk | Low risk | Low risk | High risk | High risk | High risk | Moderate |
| Jung, 2015 (52) | | Low risk | Low risk | Low risk | Low risk | Unclear | Low risk | High risk | Moderate |
| Krumbeck, 2018 (53) | | Unclear | Unclear | Unclear | Low risk | High risk | Low risk | High risk | Low |
| Lim, 2020 (54) | | Unclear | Unclear | Unclear | Low risk | High risk | Low risk | Low risk | Low |
| Madjd, 2016 (23) | | Low risk | Low risk | Unclear | Low risk | Low risk | Low risk | Low risk | Moderate |
| Naito, 2017 (55) | | Low risk | Low risk | Low risk | Low risk | Low risk | Unclear | High risk | High |
| Nasiri 2021 (56) | | Low risk | Low risk | Low risk | Low risk | High risk | Low risk | Low risk | Moderate |
| Orak, 2022 (28) | | Unclear | Unclear | Unclear | Unclear | Low risk | High risk | High risk | Low |
| Othman 2023 (29) | | High risk | High risk | High risk | High risk | Low risk | Unclear | Low risk | Low |
| Rahayu 2021 (62) | | Unclear | Unclear | Low risk | Low risk | Unclear | Low risk | High risk | Low |
| Rajkumar, 2014 (63) | | Unclear | Unclear | High risk | Low risk | Unclear | Low risk | Unclear | Low |
| Razmpoosh, 2019 (64) | | Low risk | Low risk | Unclear | Unclear | High risk | High risk | High risk | Low |
| Schellekens, 2020 (57) | | Low risk | Low risk | Low risk | Low risk | Low risk | Low risk | Low risk | High |
| Sohn 2022 (a) (34) | | Unclear | Unclear | Unclear | Unclear | Low risk | Low risk | Low risk | Low |
| Sohn 2022 (b) (32) | | Low risk | Low risk | Unclear | Unclear | High risk | Low risk | Low risk | Low |
| Stenman, 2016 (22) | | Low risk | Low risk | Low risk | Low risk | High risk | Low risk | Low risk | Moderate |
| Toshimitsu, 2022 (35) | | Low risk | Low risk | Low risk | Low risk | Low risk | Low risk | High risk | High |
| Zarrati, 2014 (58) and Zarrati, 2013 (59) | | Low risk | Low risk | Low risk | Low risk | Low risk | Unclear | Low risk | High |
| * Main domain | | | | | | | | | |

Supp Figure 1. Forest plot displaying subgroup analysis of the outcome of systolic blood pressure based on age of participants

Supp Figure 2. Forest plot displaying subgroup analysis of the outcome of systolic blood pressure based on gender of participants

Supp Figure 3. Forest plot displaying subgroup analysis of the outcome of systolic blood pressure based on health status of participants

Supp Figure 4. Forest plot displaying subgroup analysis of the outcome of systolic blood pressure based on body mass index of participants

Supp Figure 5. Forest plot displaying subgroup analysis of the outcome of systolic blood pressure based on probiotic type

Supp Figure 6. Forest plot displaying subgroup analysis of the outcome of systolic blood pressure based on probiotic dosage

Supp Figure 7. Forest plot displaying subgroup analysis of the outcome of systolic blood pressure based on duration of treatment

Supp Figure 8. Forest plot displaying subgroup analysis of the outcome of systolic blood pressure based on delivery format

Supp Figure 9. Forest plot displaying subgroup analysis of the outcome of diastolic blood pressure based on age of participants

Supp Figure 10. Forest plot displaying subgroup analysis of the outcome of diastolic blood pressure based on gender of participants

Supp Figure 11. Forest plot displaying subgroup analysis of the outcome of diastolic blood pressure based on health status of participants

Supp Figure 12. Forest plot displaying subgroup analysis of the outcome of diastolic blood pressure based on body mass index of participants

Supp Figure 13. Forest plot displaying subgroup analysis of the outcome of diastolic blood pressure based on probiotic type

Supp Figure 14. Forest plot displaying subgroup analysis of the outcome of diastolic blood pressure based on probiotic dosage

Supp Figure 15. Forest plot displaying subgroup analysis of the outcome of diastolic blood pressure based on duration of treatment

Supp Figure 16. Forest plot displaying subgroup analysis of the outcome of diastolic blood pressure based on delivery format

Supp Figure 17. Forest plot displaying subgroup analysis of the outcome of fasting plasma glucose based on age of participants

Supp Figure 18. Forest plot displaying subgroup analysis of the outcome of fasting plasma glucose based on gender of participants

Supp Figure 19. Forest plot displaying subgroup analysis of the outcome of fasting plasma glucose based on health status of participants

Supp Figure 20. Forest plot displaying subgroup analysis of the outcome of fasting plasma glucose based on body mass index of participants

Supp Figure 21. Forest plot displaying subgroup analysis of the outcome of fasting plasma glucose based on probiotic type

Supp Figure 22. Forest plot displaying subgroup analysis of the outcome of fasting plasma glucose based on treatment characteristics including probiotic dosage

Supp Figure 23. Forest plot displaying subgroup analysis of the outcome of fasting plasma glucose based on duration of treatment

Supp Figure 24. Forest plot displaying subgroup analysis of the outcome of fasting plasma glucose based on delivery format

Supp Figure 25. Forest plot displaying subgroup analysis of the outcome of Hemoglobin A1C based on age of participants

Supp Figure 26. Forest plot displaying subgroup analysis of the outcome of Hemoglobin A1C based on gender of participants

Supp Figure 27. Forest plot displaying subgroup analysis of the outcome of Hemoglobin A1C based on health status of participants

Supp Figure 28. Forest plot displaying subgroup analysis of the outcome of Hemoglobin A1C based on body mass index of participants

Supp Figure 29. Forest plot displaying subgroup analysis of the outcome of Hemoglobin A1C based on probiotic type

Supp Figure 30. Forest plot displaying subgroup analysis of the outcome of Hemoglobin A1C based on probiotic dosage

Supp Figure 31. Forest plot displaying subgroup analysis of the outcome of Hemoglobin A1C based on duration of treatment

Supp Figure 32. Forest plot displaying subgroup analysis of the outcome of Hemoglobin A1C based on delivery format

Supp Figure 33. Forest plot displaying subgroup analysis of the outcome of fasting serum insulin based on age of participants

Supp Figure 34. Forest plot displaying subgroup analysis of the outcome of fasting serum insulin based on gender of participants

Supp Figure 35. Forest plot displaying subgroup analysis of the outcome of fasting serum insulin based on health status of participants

Supp Figure 36. Forest plot displaying subgroup analysis of the outcome of fasting serum insulin based on body mass index of participants

Supp Figure 37. Forest plot displaying subgroup analysis of the outcome of fasting serum insulin based on probiotic type

Supp Figure 38. Forest plot displaying subgroup analysis of the outcome of fasting serum insulin based on probiotic dosage

Supp Figure 39. Forest plot displaying subgroup analysis of the outcome of fasting serum insulin based on duration of treatment

Supp Figure 40. Forest plot displaying subgroup analysis of the outcome of fasting serum insulin based on delivery format.

Supp Figure 41. Forest plot displaying subgroup analysis of the outcome of total cholesterol based on age of participants

Supp Figure 42. Forest plot displaying subgroup analysis of the outcome of total cholesterol based on gender of participants

Supp Figure 43. Forest plot displaying subgroup analysis of the outcome of total cholesterol based on health status of participants

Supp Figure 44. Forest plot displaying subgroup analysis of the outcome of total cholesterol based on body mass index of participants

Supp Figure 45. Forest plot displaying subgroup analysis of the outcome of total cholesterol based on probiotic type

Supp Figure 46. Forest plot displaying subgroup analysis of the outcome of total cholesterol based on probiotic dosage

Supp Figure 47. Forest plot displaying subgroup analysis of the outcome of total cholesterol based on duration of treatment

Supp Figure 48. Forest plot displaying subgroup analysis of the outcome of total cholesterol based on delivery format.

Supp Figure 49. Forest plot displaying subgroup analysis of the outcome of low-density lipoprotein cholesterol based on age of participants

Supp Figure 50. Forest plot displaying subgroup analysis of the outcome of low-density lipoprotein cholesterol based on gender of participants

Supp Figure 51. Forest plot displaying subgroup analysis of the outcome of low-density lipoprotein cholesterol based on health status of participants

Supp Figure 52. Forest plot displaying subgroup analysis of the outcome of low-density lipoprotein cholesterol based on body mass index of participants

Supp Figure 53. Forest plot displaying subgroup analysis of the outcome of low-density lipoprotein cholesterol based on probiotic type

Supp Figure 54. Forest plot displaying subgroup analysis of the outcome of low-density lipoprotein cholesterol based on probiotic dosage

Supp Figure 55. Forest plot displaying subgroup analysis of the outcome of low-density lipoprotein cholesterol based on duration of treatment

Supp Figure 56. Forest plot displaying subgroup analysis of the outcome of low-density lipoprotein cholesterol based on delivery format.

Supp Figure 57. Forest plot displaying subgroup analysis of the outcome of high-density lipoprotein cholesterol based on age of participants

Supp Figure 58. Forest plot displaying subgroup analysis of the outcome of high-density lipoprotein cholesterol based on gender of participants

Supp Figure 59. Forest plot displaying subgroup analysis of the outcome of high-density lipoprotein cholesterol based on health status of participants

Supp Figure 60. Forest plot displaying subgroup analysis of the outcome of high-density lipoprotein cholesterol based on body mass index of participants

Supp Figure 61. Forest plot displaying subgroup analysis of the outcome of high-density lipoprotein cholesterol based on probiotic type

Supp Figure 62. Forest plot displaying subgroup analysis of the outcome of high-density lipoprotein cholesterol based on probiotic dosage

Supp Figure 63. Forest plot displaying subgroup analysis of the outcome of high-density lipoprotein cholesterol based on duration of treatment

Supp Figure 64. Forest plot displaying subgroup analysis of the outcome of high-density lipoprotein cholesterol based on delivery format.

Supp Figure 65. Forest plot displaying subgroup analysis of the outcome of serum triglyceride based on age of participants

Supp Figure 66. Forest plot displaying subgroup analysis of the outcome of serum triglyceride based on gender of participants

Supp Figure 67. Forest plot displaying subgroup analysis of the outcome of serum triglyceride based on health status of participants

Supp Figure 68. Forest plot displaying subgroup analysis of the outcome of serum triglyceride based on body mass index of participants

Supp Figure 69. Forest plot displaying subgroup analysis of the outcome of serum triglyceride based on probiotic type

Supp Figure 70. Forest plot displaying subgroup analysis of the outcome of serum triglyceride based on probiotic dosage

Supp Figure 71. Forest plot displaying subgroup analysis of the outcome of serum triglyceride based on duration of treatment

Supp Figure 72. Forest plot displaying subgroup analysis of the outcome of serum triglyceride based on delivery format

Supp Figure 73. Forest plot displaying subgroup analysis of the outcome of C-reactive protein based on age of participants

Supp Figure 74. Forest plot displaying subgroup analysis of the outcome of C-reactive protein based on gender of participants

Supp Figure 75. Forest plot displaying subgroup analysis of the outcome of C-reactive protein based on health status of participants

Supp Figure 76. Forest plot displaying subgroup analysis of the outcome of C-reactive protein based on body mass index of participants

Supp Figure 77. Forest plot displaying subgroup analysis of the outcome of C-reactive protein based on probiotic type

Supp Figure 78. Forest plot displaying subgroup analysis of the outcome of C-reactive protein based on probiotic dosage

Supp Figure 79. Forest plot displaying subgroup analysis of the outcome of C-reactive protein based on duration of treatment

Supp Figure 80. Forest plot displaying subgroup analysis of the outcome of C-reactive protein based on delivery format

Supp Figure 81. Meta-regression analysis of the (a) systolic and (b) diastolic blood pressure based on supplementation dosage.


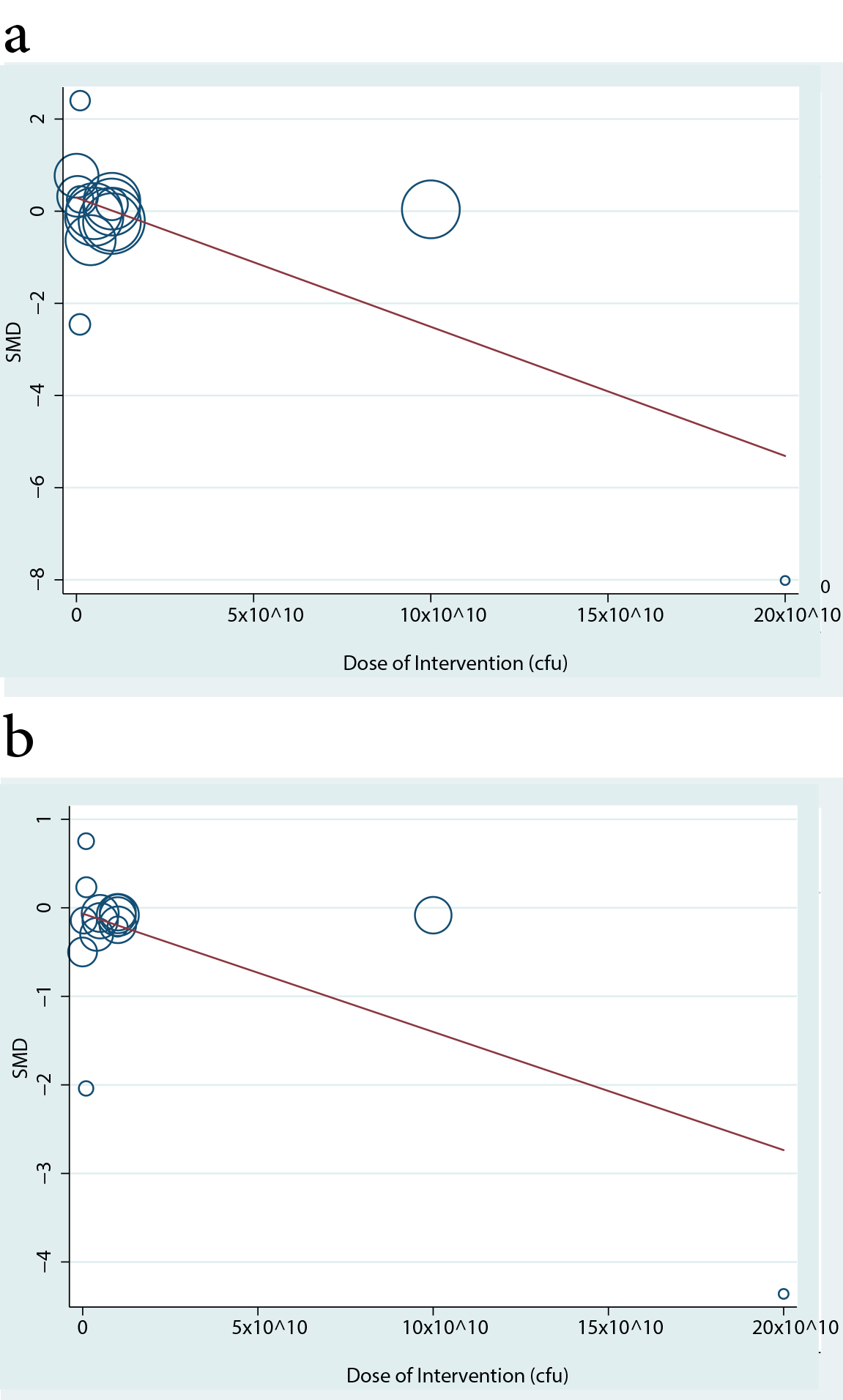


Supp Figure 82. Meta-regression analysis of the C-reactive protein based on supplementation dosage.


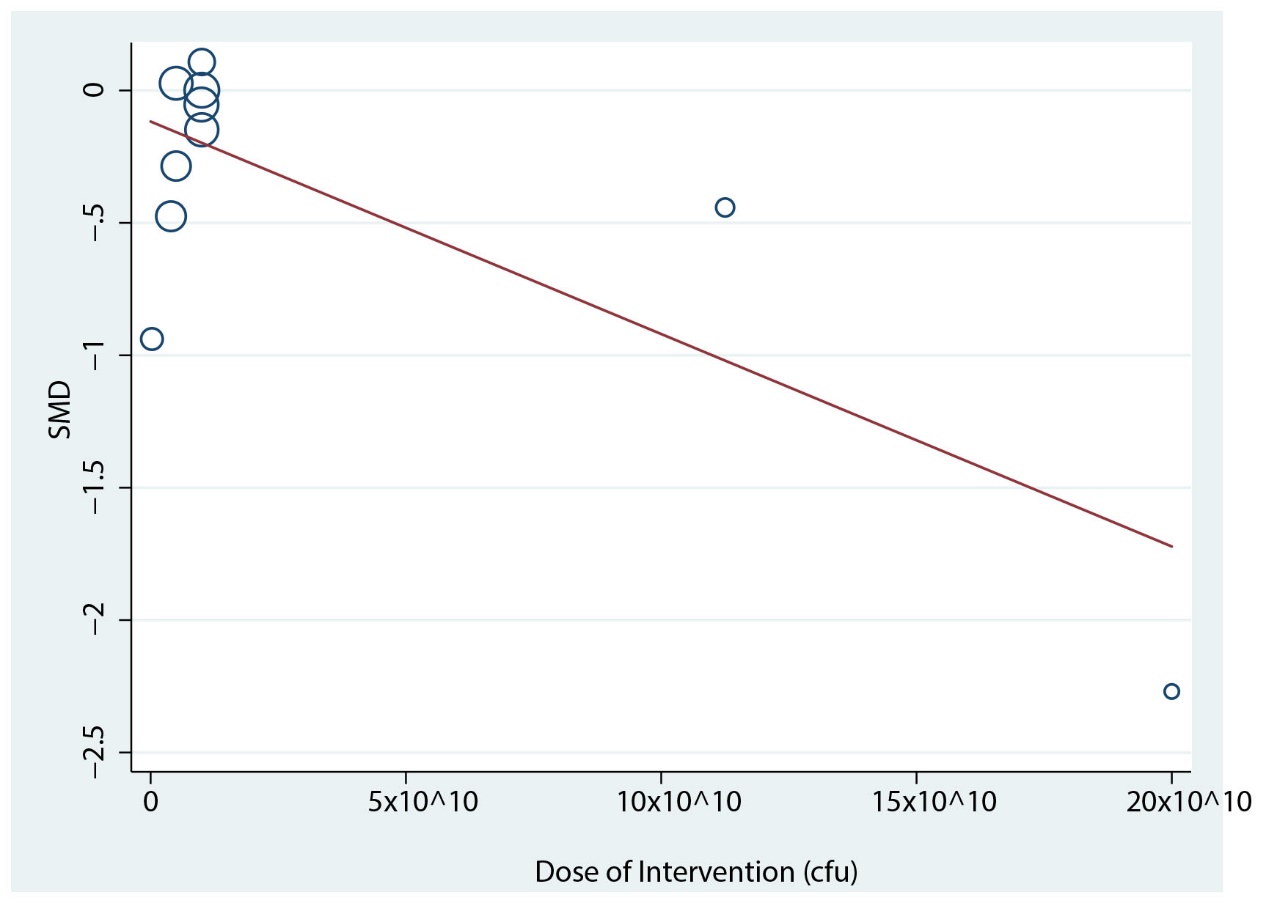


Supp Figure 83. Funnel plot displaying the publication bias of the included trials reporting the effects of probiotics on (a) systolic and (b) diastolic blood pressure.


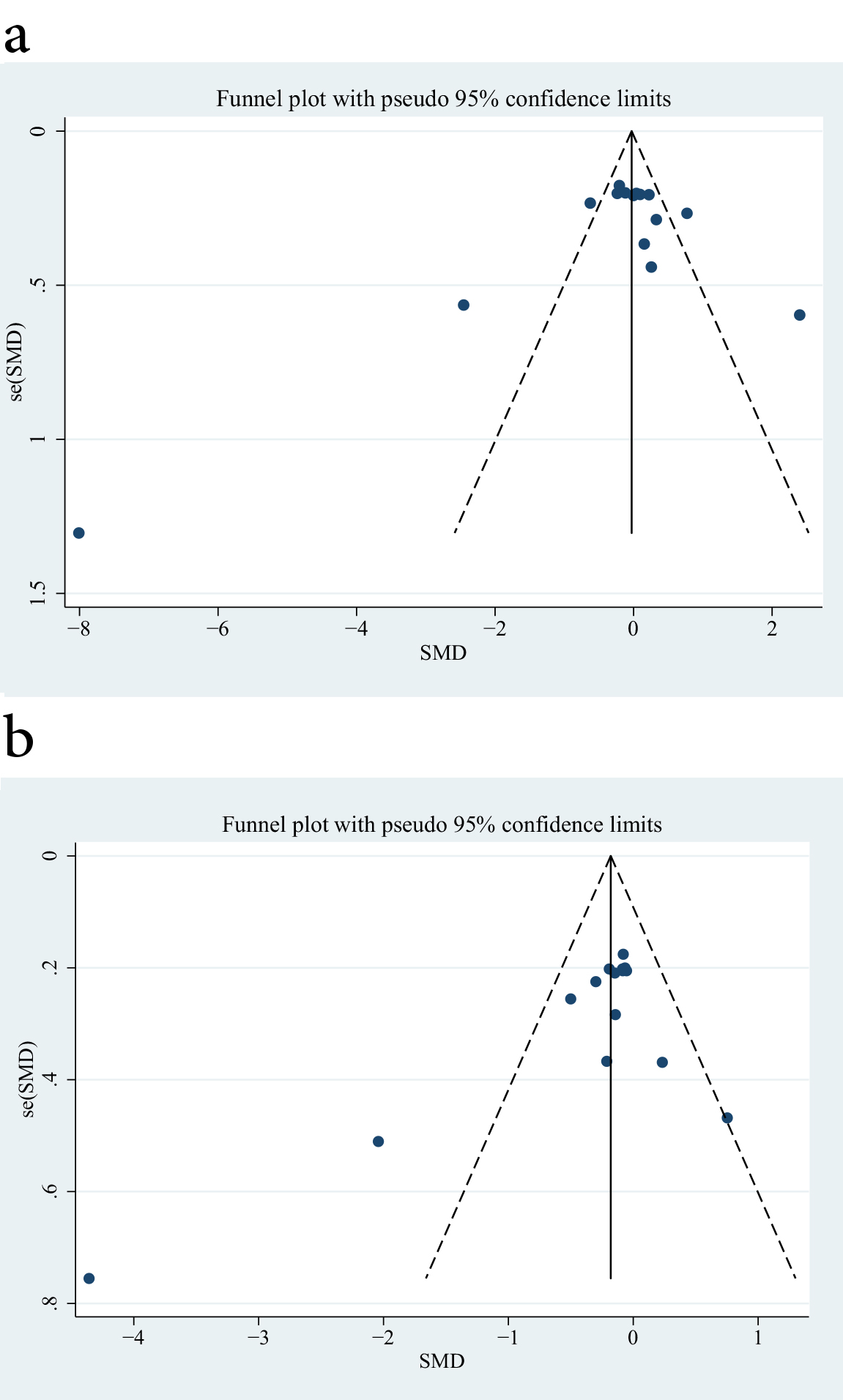


Supp Figure 84. Funnel plot displaying the publication bias of the included trials reporting the effects of probiotics on (a) fasting plasma glucose, (b) Hemoglobin A1C, and (c) fasting insulin.


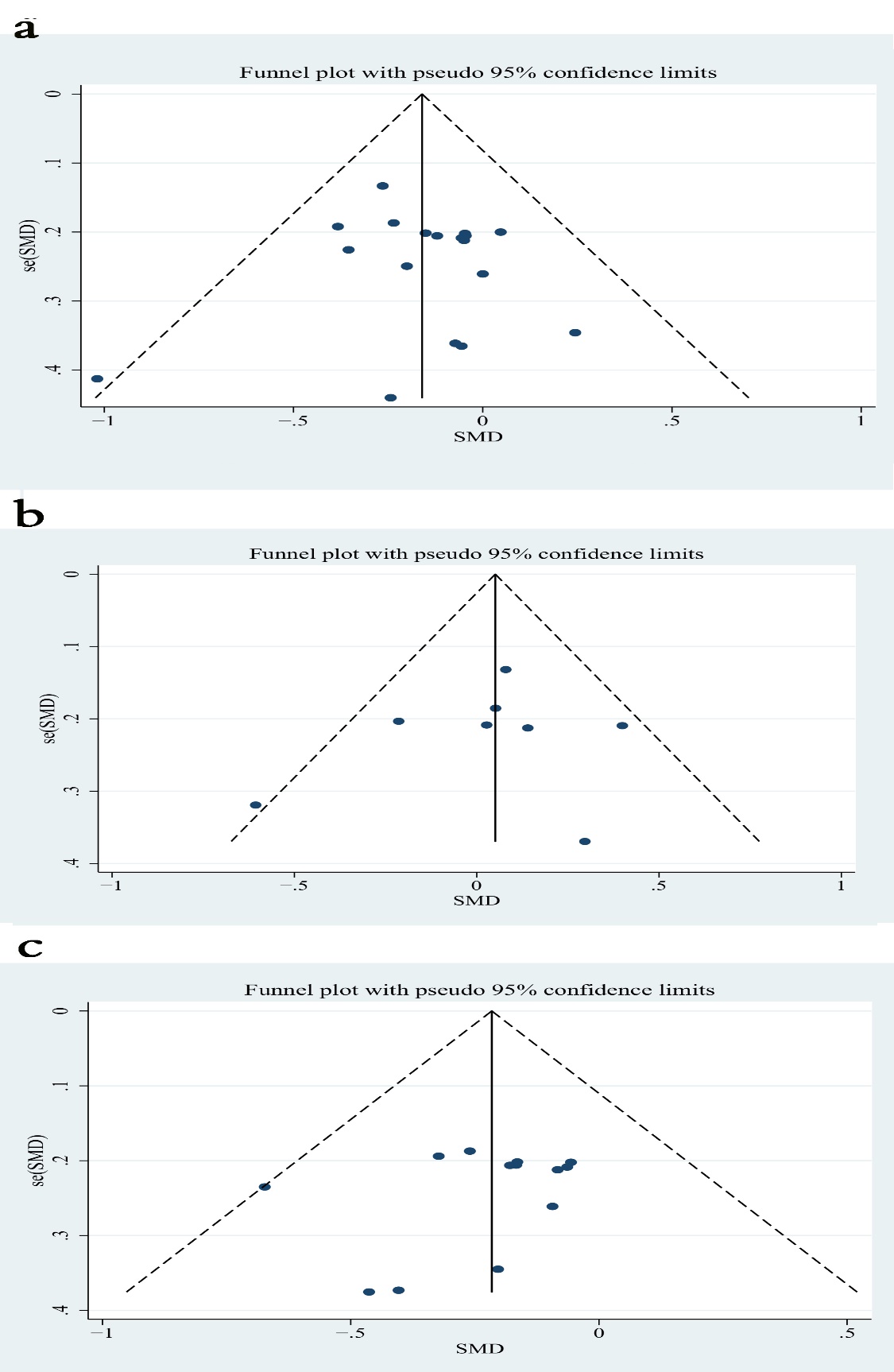


Supp Figure 85. Funnel plot displaying the publication bias of the included trials reporting the effects of probiotics on (a) total cholesterol; (b) low-density lipoprotein; (c) high-density lipoprotein; (d) triglyceride.


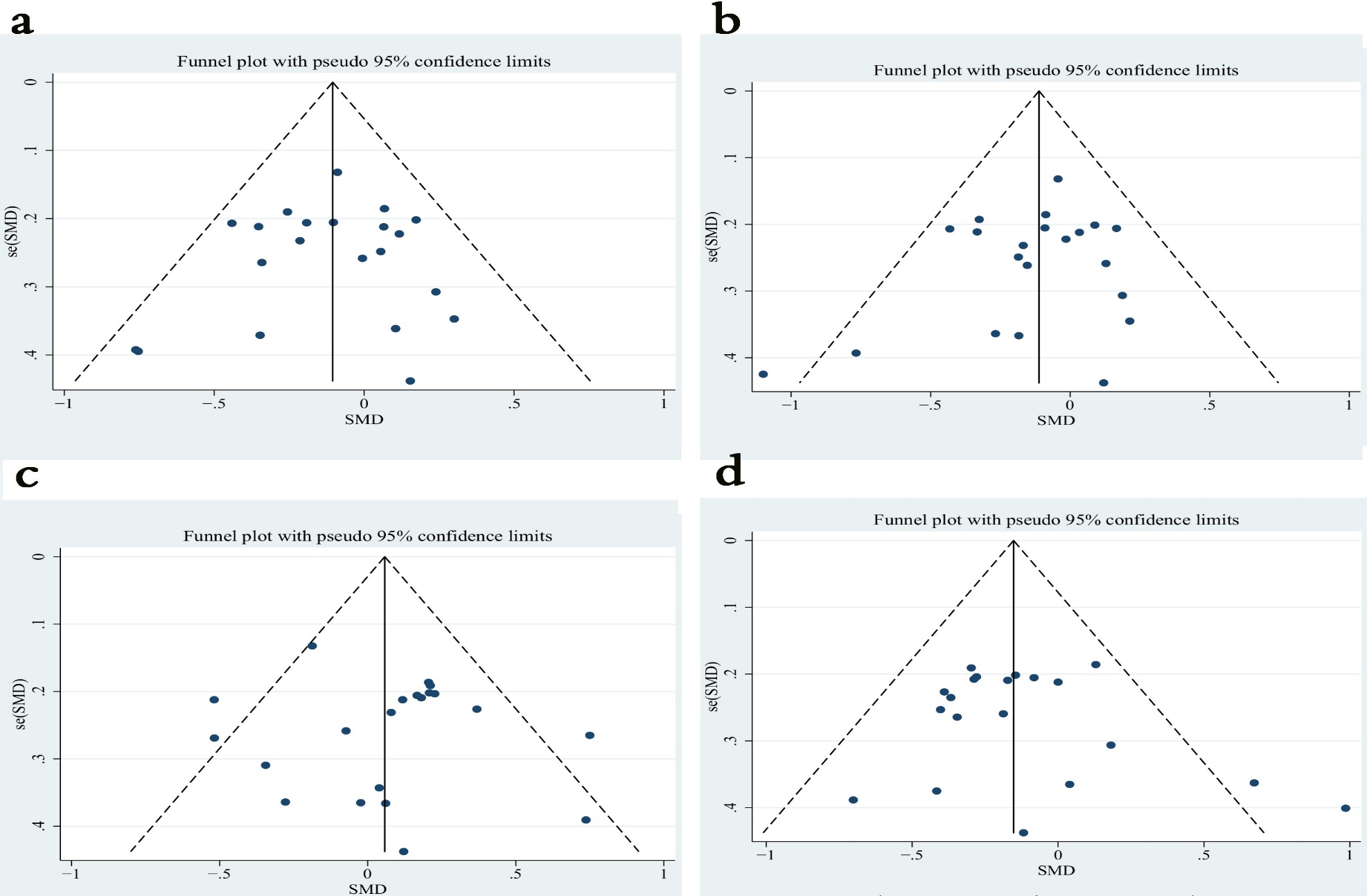


Supp Figure 86. Funnel plot displaying the publication bias of the included trials reporting the effects of probiotics on C-reactive protein.


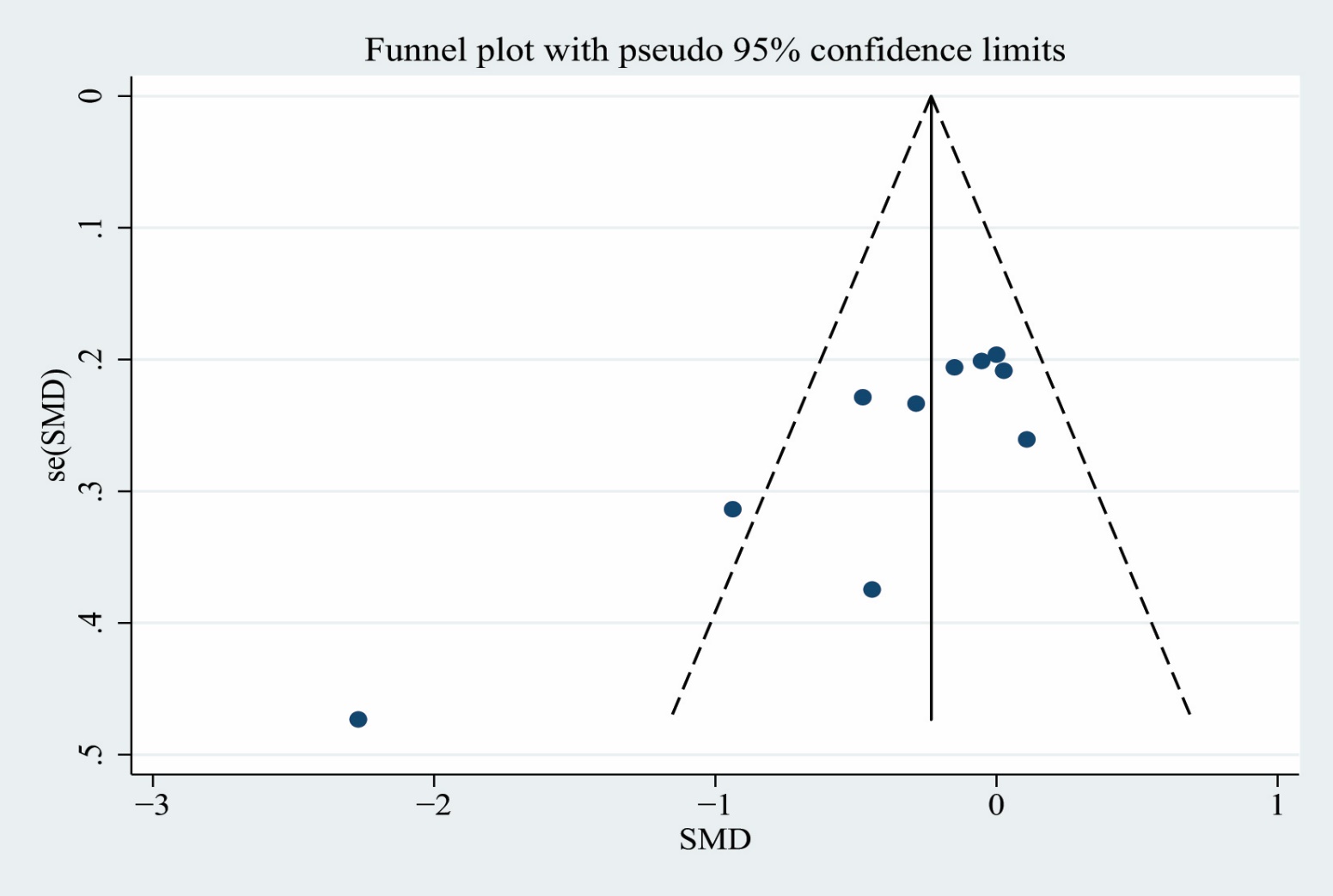

Supplement: Supplementary file 1 — TABLE S1. Electronic search strategy. TABLE S2. Primary studies' quality (risk of bias). FIGURE S1–S8. Forest plot displaying subgroup analysis of the outcome of systolic blood pressure based on participants' and treatment characteristics. FIGURE S9–S16. Forest plot displaying subgroup analysis of the outcome of diastolic blood pressure based on participants' and treatment characteristics. FIGURE S17–S24. Forest plot displaying subgroup analysis of the outcome of fasting plasma glucose based on participants' and treatment characteristics. FIGURE S25–S32. Forest plot displaying subgroup analysis of the outcome of hemoglobin A1C based on participants' and treatment characteristics. FIGURE S33–S40. Forest plot displaying subgroup analysis of the outcome of fasting serum insulin based on participants' and treatment characteristics. FIGURE S41–S48. Forest plot displaying subgroup analysis of the outcome of total cholesterol based on participants' and treatment characteristics. FIGURE S49–S56. Forest plot displaying subgroup analysis of the outcome of low‐density lipoprotein cholesterol based on participants' and treatment characteristics. FIGURE S57–S64. Forest plot displaying subgroup analysis of the outcome of high‐density lipoprotein cholesterol based on participants' and treatment characteristics. FIGURE S65–S72. Forest plot displaying subgroup analysis of the outcome of serum triglyceride based on participants' and treatment characteristics. FIGURE S73–S80. Forest plot displaying subgroup analysis of the outcome of C‐reactive protein based on participants' and treatment characteristics. FIGURE S81. Meta‐regression analysis of the (a) systolic and (b) diastolic blood pressure based on supplementation dosage. FIGURE S82. Meta‐regression analysis of the C‐reactive protein based on supplementation dosage. FIGURE S83. Funnel plot displaying the publication bias of the included trials reporting the effects of probiotics on (a) systolic and (b) diastolic blood pressure. FI [file FSN3-13-e70434-s001.docx]
